# S-Scheme charge transfer in topotactically transformed LTO/g-C_3_N_4_ for photo-Fenton mineralization in complex water matrices

**DOI:** 10.1039/d6ra06898g

**Published:** 2026-10-14

**Authors:** Fowzia S. Alamro, Wafaa B. Elsharkawy, Arafat Toghan, Ashraf A. Mohamed, Ayman M. Mostafa, Mohamed Farag, Hoda A. Ahmed, Mohamed A. Ahmed, Mahmoud A. Elsayed

**Affiliations:** a Department of Chemistry, College of Science, Princess Nourah bint Abdulrahman University P.O. Box 84428 Riyadh 11671 Saudi Arabia; b Department of Physics, College of Science and Humanities, Prince Sattam Bin Abdulaziz University P.O. Box 173 11942 Al-Kharj Saudi Arabia; c Chemistry Department, College of Science, Imam Mohammad Ibn Saud Islamic University (IMSIU) 11623 Riyadh Saudi Arabia; d Chemistry Department, Faculty of Science, Ain Shams University Cairo 11566 Egypt mahmoud.a.elsayed1552@yahoo.com; e Department of Chemistry, College of Science in Yanbu, Taibah University Yanbu Governorate Saudi Arabia; f Department of Physics, College of Science, Qassim University P.O.Box 6644 Buraydah Almolaydah 51452 Saudi Arabia; g The Higher Institute for Optics Technology, Culture and Science City Cairo 17361 Egypt

## Abstract

The development of visible-light-driven photo-Fenton catalysts for the mineralization of recalcitrant organic pollutants remains a challenge in wastewater treatment. In this study, an S-scheme-type ZnFe_2_O_4_/ZnO/CuO@g-C_3_N_4_ (GLTO) heterojunction was prepared *via* topotactic transformation of a Zn–Cu–Fe layered triple hydroxide (LTH) precursor, followed by ultrasonic assembly with exfoliated g-C_3_N_4_. Structural and optical characterizations revealed intimate interfacial contact, a mesoporous architecture (mean pore diameter, 31.0 nm; specific surface area, 49.6 m^2^ g^−1^), and an optical band gap of 2.4 eV. The GLTO composite demonstrated visible-light photo-Fenton activity for the degradation of rhodamine B dye (RhB), achieving 93.5% dye removal and 65% total organic carbon (TOC) reduction within 28 min. Liquid chromatography-mass spectrometry revealed progressive conversion to low-molecular-weight products, with the detected intermediates tentatively assigned. The apparent rate constant (0.0877 min^−1^) was ∼30-fold higher than that of bare LTO. The catalyst maintained >90% degradation efficiency at 10 mM NaCl and 72% degradation at 10 mg L^−1^ humic acid under the tested model water conditions, and retained >95% of its initial activity after five cycles. Electrochemical impedance spectroscopy and electron spin resonance spectroscopy supported enhanced charge separation and the involvement of reactive oxygen species, consistent with the proposed S-scheme mechanism. In this model, electrons are retained on g-C_3_N_4_, while holes are retained on the LTO component. ECOSAR modeling predicted a progressive reduction in the potential ecotoxicity of the detected intermediates. These findings demonstrate the potential of LTH-derived multimetal oxides coupled with g-C_3_N_4_ for the photo-Fenton treatment of organic pollutants under the tested model water conditions.

## Abstract 2

The development of visible-light-driven photo-Fenton catalysts for the mineralization of recalcitrant organic pollutants remains a challenge in wastewater treatment.

## Graphical abstract

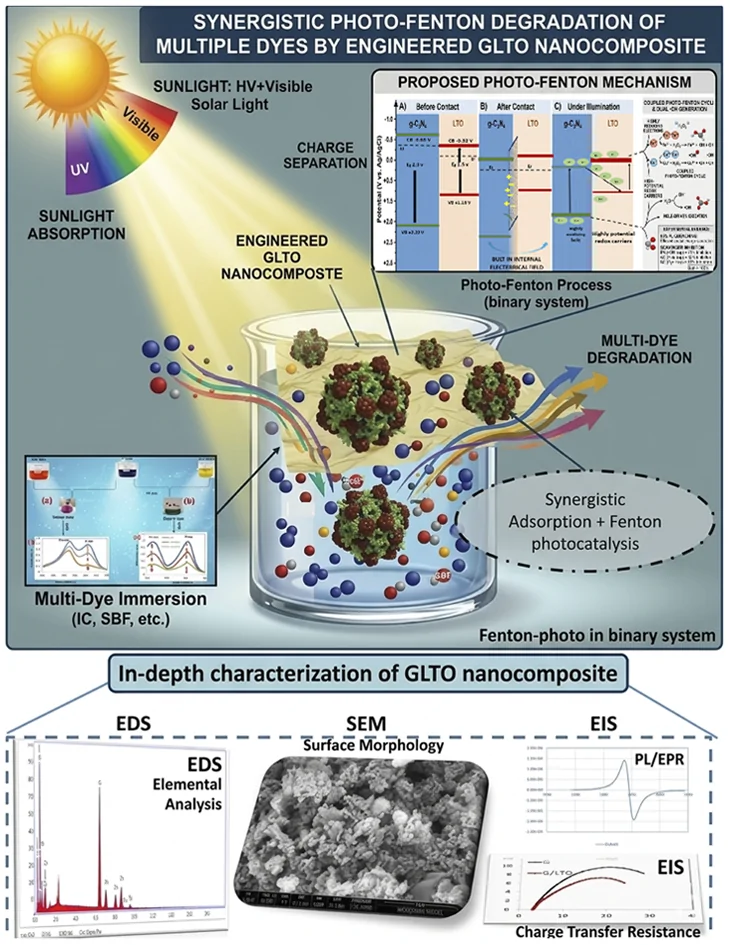

## 1. Introduction

The global textile industry consumes approximately 10 million tons of synthetic dyes annually, with an estimated 15% discharged as untreated effluent into receiving water bodies.^1,2^ Among more than 100 000 commercially available dyes, rhodamine B (RhB, C_28_H_31_ClN_2_O_3_, molecular weight 479 g mol^−1^) is a prominent pollutant due to its widespread application in the textile, leather, and paper industries, as well as its use as a fluorescent tracer.^3–5^ The xanthene structure of RhB, which includes diethylamino substituents, confers high chemical stability, low biodegradability, and environmental persistence.^6^ Furthermore, RhB exhibits toxicity toward aquatic organisms, with lethal concentration (LC_50_) values reported in the low mg L^−1^ range for species such as zebrafish and *Daphnia magna*. Because of its documented toxicity and potential carcinogenic effects, its application in the food industry is prohibited in multiple jurisdictions, including the European Union and China. Conventional remediation strategies-such as adsorption, coagulation–flocculation, membrane filtration, and biological treatment-exhibit inherent limitations.^40–42^ These methods frequently transfer pollutants between phases, generate secondary sludge, require substantial operational costs, or fail to achieve substantial mineralization, which can result in the formation of toxic intermediates.^7,8^

Advanced oxidation processes (AOPs) address these limitations by generating highly reactive oxygen species, primarily hydroxyl radicals (˙OH, *E*° ≈ 2.80 V), which non-selectively degrade organic molecules into CO_2_, H_2_O, and inorganic ions. Common AOPs include (i) ozonation (O_3_/OH^−^, O_3_/H_2_O_2_), (ii) photocatalysis (TiO_2_/UV, g-C_3_N_4_/Vis), (iii) electrochemical oxidation, and (iv) Fenton-based reactions (Fe^2+^/H_2_O_2_).^9–13^ However, each method has specific constraints. Ozonation can produce bromate and other toxic disinfection by-products in bromide-containing waters. Photocatalysis using wide-bandgap semiconductors, such as TiO_2_ (*E*_g_ = 3.2 eV), requires UV irradiation, restricting solar energy utilization to approximately 5%. Electrochemical methods involve high electrode costs and are often constrained by mass transport limitations.^14,15^ Furthermore, the classical homogeneous Fenton process (Fe^2+^ + H_2_O_2_ → Fe^3+^ + ˙OH + OH^−^) is optimal only under highly acidic conditions (pH 2.5–3.5), generates substantial iron sludge, and complicates catalyst recovery.^16,17^ To overcome these challenges, heterogeneous photo-Fenton systems integrate photocatalysts with Fenton-active metals (*e.g.*, Fe, Cu, Co) immobilized on solid supports. This approach enables operation at near-neutral pH, facilitates catalyst recycling, and utilizes light irradiation to accelerate the rate-limiting Fe^3+^/Fe^2+^ reduction cycle.

Graphitic carbon nitride (g-C_3_N_4_) is recognized as an effective visible-light photocatalyst for such systems.^18^ Its tri-*s*-triazine framework provides a band gap of 2.7–2.9 eV (absorption edge ∼450 nm) and a conduction band (CB) ranging from −1.1 to −1.3 V, which is sufficiently negative to reduce Fe^3+^ to Fe^2+^ and O_2_ to ˙O_2_^−^. Furthermore, it demonstrates considerable chemical stability.^19^ However, pristine g-C_3_N_4_ is limited by rapid charge recombination (evidenced by strong photoluminescence) and a low specific surface area (10–30 m^2^ g^−1^ due to sheet stacking). Additionally, its valence band (VB) position (+1.6 to +1.8 V) is thermodynamically insufficient for direct ˙OH generation from H_2_O (*E*°(H_2_O/˙OH) = +2.40 V).^20,21^ Consequently, pristine g-C_3_N_4_ exhibits limited photo-Fenton efficiency. The design of heterojunctions has evolved from conventional Type-II structures toward more advanced charge-transfer mechanisms. In a conventional Type-II heterojunction, electrons migrate to the less negative conduction band and holes to the less positive valence band, which spatially separates carriers but sacrifices the overall redox potential of the system.^22,23^ In contrast, S-scheme (step-scheme) heterojunctions integrate a reduction photocatalyst (with a more negative CB) and an oxidation photocatalyst (with a more positive VB). This configuration exploits an internal electric field at the interface to recombine the weaker photogenerated carriers-electrons from the oxidation component and holes from the reduction component. Consequently, it preserves the strongly reducing electrons in the reduction component and the strongly oxidizing holes in the oxidation component.^24,25^ This architecture is highly advantageous for photo-Fenton applications, as the preserved electrons can continuously reduce Fe^3+^/Cu^2+^ to sustain the Fenton cycle, while the preserved holes can directly degrade organic pollutants or oxidize water and hydroxyl ions.

Layered triple hydroxides (LTHs) represent a class of anionic clays in which three distinct divalent or trivalent metal cations occupy the octahedral sites of brucite-like layers, with interlayer anions (such as carbonate, nitrate, or chloride) balancing the residual positive charge.^26,27^ This structural feature distinguishes LTHs from conventional layered double hydroxides (LDHs), which incorporate only two metal cation species. The integration of three distinct metals provides enhanced compositional flexibility. By selecting combinations that pair redox-active transition metals (*e.g.*, Fe, Cu, Mn, Co) with structural stabilizers (*e.g.*, Zn, Al, Mg), LTH precursors can be specifically tailored for targeted catalytic, magnetic, or electronic applications.^26^ Furthermore, the layered architecture provides a substantial interlayer space capable of accommodating various anions, while the hydroxyl-rich framework facilitates subsequent topotactic transformations.

Upon thermal treatment, typically between 400 and 600 °C, LTHs undergo a topotactic transformation into LTH-derived multimetal oxides.^26,28^ Unlike conventional synthetic methods that often suffer from severe nanoparticle agglomeration, this *in situ* calcination induces the de-hydroxylation of metal hydroxide layers and the decomposition of interlayer anions while strictly preserving the precursor's lamellar morphology. Consequently, the resulting LTH-derived oxides exhibit structural advantages critical for advanced catalysis. The release of interlayer gases yields highly mesoporous structures with large specific surface areas and abundant oxygen vacancies. Concurrently, the topotactic nature of the transformation ensures the atomic-level dispersion of the three metal species within the oxide matrix, effectively suppressing phase segregation. This uniform dispersion promotes the formation of intimate heterojunction interfaces between the distinct oxide phases, which significantly enhances charge separation. Furthermore, the structural memory effect of the LTH precursor stabilizes mixed-valence transition metal pairs (*e.g.*, Fe^3+^/Fe^2+^, Cu^2+^/Cu^+^) at the catalytic surface, providing the essential active sites required to drive continuous Fenton-like redox cycles.^29,30^

The integration of the LTO with exfoliated g-C_3_N_4_ enhances the overall photo-Fenton catalytic performance. With its more negative conduction band, g-C_3_N_4_ functions as an effective electron donor within the proposed heterojunction.^31^ Upon interfacial contact, charge redistribution and interfacial band bending are proposed to establish an internal electric field that favors selective recombination of the less energetically favorable photogenerated carriers. Under visible-light irradiation, this proposed S-scheme pathway involves recombination of electrons in the LTO component with holes in g-C_3_N_4_, thereby preserving the strongly reducing electrons in the conduction band of g-C_3_N_4_ and the oxidizing holes in the valence band of LTO.^31,32^ The retained electrons on g-C_3_N_4_ can participate in the reduction of transition-metal centers (*e.g.*, Fe^3+^/Fe^2+^ and Cu^2+^/Cu^+^), thereby contributing to H_2_O_2_ activation and the associated Fenton-like reactions, while the holes retained in LTO can contribute to the surface oxidation of adsorbed organic species.^32,33^

In this study, a visible-light-driven photo-Fenton catalyst based on a ZnFe_2_O_4_/ZnO/CuO@g-C_3_N_4_ (GLTO) heterojunction was synthesized *via* topotactic transformation of a Zn–Cu–Fe layered triple hydroxide (LTH) precursor, followed by ultrasonic assembly with exfoliated g-C_3_N_4_. The redox-active Fe and Cu centers, together with the photogenerated electrons retained in the g-C_3_N_4_ conduction band, can contribute to Fe/Cu redox cycling and H_2_O_2_ activation. Photoluminescence quenching, electrochemical impedance spectroscopy, and electron spin resonance measurements provided supporting evidence for enhanced charge separation and the involvement of reactive oxygen species, consistent with the proposed S-scheme charge-transfer pathway. A stepwise degradation pathway for rhodamine B was proposed based on LC-MS analysis, and the potential toxicity of the detected intermediates was assessed using ECOSAR modeling. The results demonstrate the applicability of the GLTO heterojunction for visible-light photo-Fenton degradation of organic pollutants under the tested model water conditions, including NaCl- and humic-acid-containing solutions.

## 2. Experimental

### 2.1 Materials and reagents

#### 2.1.1 Synthesis of exfoliated g-C_3_N_4_ nanosheets

The fabrication of exfoliated g-C_3_N_4_ nanosheets was initiated through the thermal polymerization of a nitrogen-rich precursor to form the bulk graphitic structure. Initially, a predetermined amount of urea was placed in a covered alumina crucible and subjected to calcination in a muffle furnace. The temperature was increased at a steady heating rate of 5 °C min^−1^ to 550 °C, where it was maintained for 4 hours to ensure the complete condensation of the heptazine units. After cooling to room temperature, the resulting yellow bulk g-C_3_N_4_ was ground into a fine powder. To achieve the transition from the bulk material to ultrathin nanosheets, a liquid-phase exfoliation (LPE) strategy was employed. The bulk powder was dispersed in a mixture of deionized water and ethanol to diminish the interlayer van der Waals forces. This suspension was then subjected to high-intensity ultrasonic probe sonication for 6 hours, while being maintained in an ice bath to prevent thermal degradation and aggregation. The mechanical energy generated by acoustic cavitation effectively exfoliated the bulk material into few-layer nanosheets. Finally, the exfoliated product was isolated *via* high-speed centrifugation, washed repeatedly with deionized water and ethanol to remove any residual unreacted species, and dried to preserve the high-surface-area nanosheet morphology.

#### 2.1.2 Fabrication of ZnFe_2_O_4_/ZnO/CuO (LTO)

The LTO ternary heterojunction particles were fabricated by first synthesizing a Zn–Cu–Fe layered triple hydroxide (LTH) precursor *via* a carefully controlled co-precipitation method. Stoichiometric quantities of zinc acetate dihydrate, copper(ii) sulfate pentahydrate, and iron(iii) chloride were dissolved in ultrapure deionized water to achieve a total cation concentration of 0.25 M with a molar ratio of 2.5 : 0.5 : 1.0 (Zn : Cu : Fe). This precursor solution was introduced dropwise into a reaction vessel containing a vigorously stirred alkaline solution of 1.0 M NaOH and Na_2_CO_3_, which maintained a stable pH of 10.0 and provided the necessary carbonate ions for the LTH interlayer. To optimize crystallinity and promote Ostwald ripening, the resulting suspension underwent a two-step aging protocol: an initial 12 hours thermal treatment at 80 °C under continuous stirring, followed by a 48 hours static aging period at room temperature. The matured LTH precipitate was recovered *via* vacuum filtration and washed repeatedly with deionized water and absolute ethanol until a neutral pH was reached and the absence of sulfate ions was confirmed using a BaCl_2_ test. Following drying at 80 °C for 12 hours, the LTH precursor was subjected to thermal decomposition in a programmable tube furnace. Calcination was conducted at 500 °C for 2 hours under a static air atmosphere with a heating rate of 3 °C min^−1^, facilitating gradual de-hydroxylation and topotactic phase transformation into the final crystalline multi-metal oxide heterojunction (Fig. 1).

**Fig. 1:**
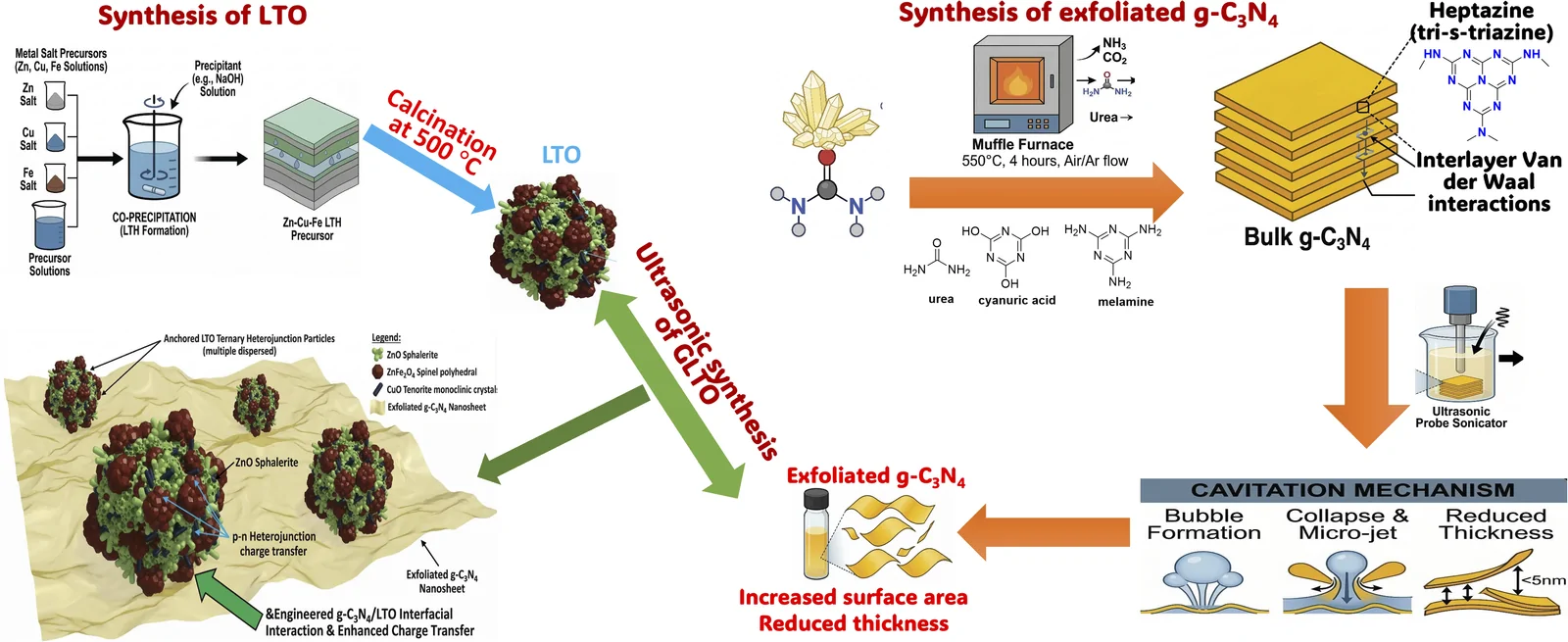
A schematic representation of the synthesis of GLTO nanocomposite.

#### 2.1.3 Fabrication of GLTO

The GLTO nanocomposites were synthesized by anchoring the previously prepared LTO ternary heterojunction particles onto the surface of exfoliated g-C_3_N_4_ nanosheets *via* an ultrasonic-assisted assembly strategy. To evaluate the impact of heterojunction loading on catalytic performance, three distinct composites were fabricated with LTO mass percentages of 5%, 10%, and 20% relative to the g-C_3_N_4_ support, designated as GLTO-5, GLTO-10, and GLTO-20, respectively. In a typical procedure, a predetermined quantity of exfoliated g-C_3_N_4_ was dispersed in 100 mL of absolute ethanol and sonicated for 30 minutes to ensure a homogeneous suspension. Simultaneously, the calculated mass of LTO powder corresponding to each loading level was dispersed separately in ethanol and subsequently introduced dropwise into the g-C_3_N_4_ suspension under continuous stirring. The resulting mixture was subjected to ultrasonic treatment for 4 hours; the applied mechanical energy facilitated uniform dispersion and promoted the interfacial anchoring of the LTO clusters onto the g-C_3_N_4_ nanosheets *via* electrostatic and van der Waals interactions. Finally, the synthesized GLTO composites were isolated *via* vacuum filtration, washed repeatedly with deionized water and ethanol to remove any unattached species, and dried under vacuum at 60 °C for 12 hours to yield the final photocatalysts (Fig. 1).

## 3. Results and discussion

### 3.1 Characterization of the LTO heterostructure

The XRD patterns of g-C_3_N_4_, LTO, and the GLTO composite are presented in Fig. 2a. Exfoliated g-C_3_N_4_ exhibits two broad diffraction peaks at approximately 13.8° and 27.3°. The former corresponds to the (100) in-plane structural packing motif of the heptazine network; its broadness reflects reduced long-range order, consistent with the exfoliation into few-layer nanosheets. The latter, more prominent peak arises from the (002) interlayer stacking of the conjugated aromatic systems. Its retention confirms the structural integrity of the graphitic framework post-exfoliation, although the peak remains broader than that of bulk g-C_3_N_4_ due to reduced stacking coherence.

**Fig. 2:**
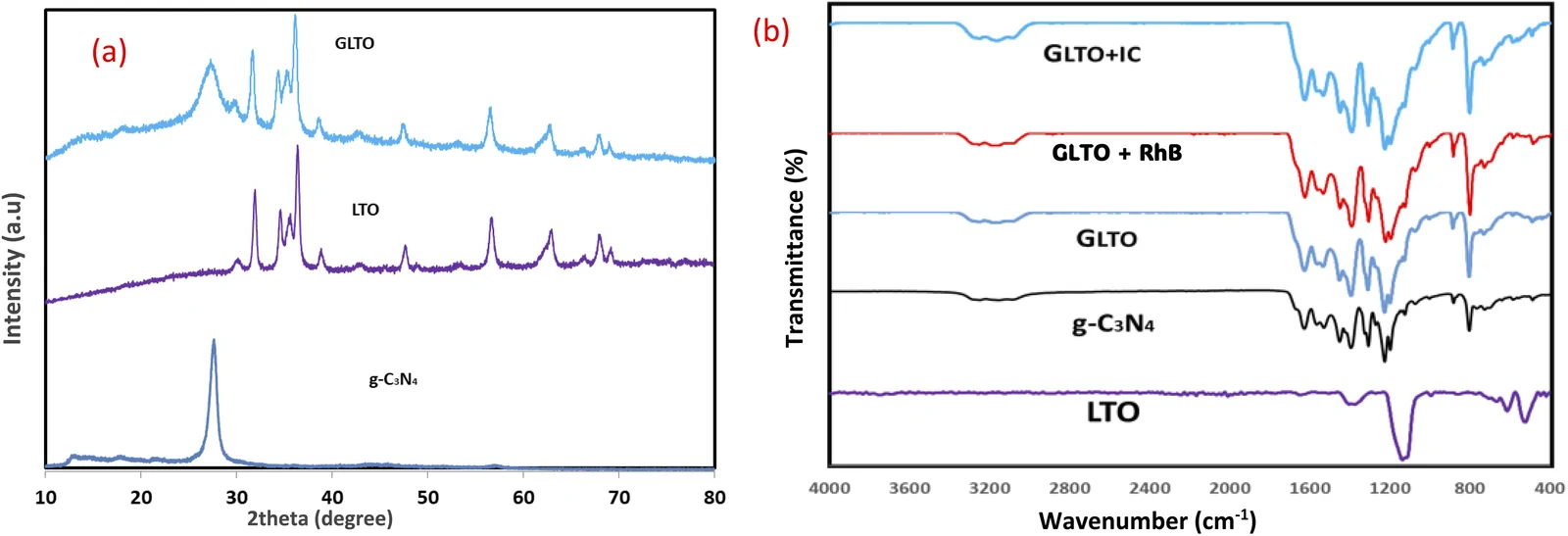
(a) XRD patterns of g-C_3_N_4_ (G), LTO, and GLTO. (b) FTIR spectra of G, LTO, and GLTO before and after adsorption of Rhodamine B (RhB) and Indigo Carmine (IC).

The pattern for the LTO composite, obtained by calcining the Zn–Cu–Fe LTH precursor, displays well-resolved reflections corresponding to three distinct crystalline phases. The dominant phase is hexagonal wurtzite ZnO, with prominent reflections at 31.92° (*d* = 2.8015 Å, FWHM = 0.382°), 34.58° (*d* = 2.5916 Å, FWHM = 0.407°), 36.40° (*d* = 2.4659 Å, FWHM = 0.376°), 47.69° (*d* = 1.9056 Å, FWHM = 0.440°), 56.68° (*d* = 1.6227 Å, FWHM = 0.502°), and 62.90° (*d* = 1.4763 Å, FWHM = 0.660°). Monoclinic CuO is identified by peaks at 35.56° (*d* = 2.5226 Å, FWHM = 0.794°), 38.82° (*d* = 2.3180 Å, FWHM = 0.480°), and 48.84° (*d* = 1.8634 Å, FWHM = 0.390°). Cubic spinel ZnFe_2_O_4_ exhibits broader reflections at 30.08° (*d* = 2.968 Å, FWHM = 0.720°), 35.56° (overlapped with CuO), 42.96° (*d* = 2.104 Å, FWHM = 0.870°), and 53.52° (*d* = 1.7107 Å, FWHM = 0.960°). No amorphous background or secondary impurity peaks were detected, indicating the complete decomposition of the LTH precursor into an intimate mixture of ZnO, CuO, and ZnFe_2_O_4_.

The GLTO heterojunction pattern superimposes the reflections of both the carbon nitride and the ternary oxides. However, a systematic shift of the LTO peaks toward lower 2θ angles is observed in the composite relative to bare LTO. For instance, the ZnO (101) peak shifts from 36.40° to 36.08° (*Δ* = −0.32°), the ZnO (100) from 31.92° to 31.59° (*Δ* = −0.33°), and the combined CuO/ZnFe_2_O_4_ peak from 35.56° to 35.24° (*Δ* = −0.32°). While minor diffraction shifts can occasionally arise from sample displacement during measurement, the systematic nature of this shift across multiple planes suggests a slight expansion in the *d*-spacing. Concurrently, a minor but measurable broadening is observed in the FWHM of the LTO peaks (*e.g.*, ZnO (101) increases from 0.376° to 0.446°). Together, this systematic shift and concurrent peak broadening suggest the presence of intimate interfacial interactions and potential microstrain induced by the ultrasonic assembly of the LTO nanoparticles onto the flexible g-C_3_N_4_ nanosheets, rather than mere physical mixing.

The FTIR spectrum of bare LTO exhibits characteristic metal–oxygen vibrations, supporting the formation of the ternary oxide heterojunction (Fig. 2b). The broad absorption band centered between 3400 and 3200 cm^−1^ corresponds to the O–H stretching of surface hydroxyl groups, indicating a hydrophilic surface amenable to aqueous interactions. Minor bands at 2920 and 2850 cm^−1^ arise from asymmetric and symmetric C–H stretching, likely originating from residual organic precursors. The band at 1630 cm^−1^ is attributed to the O–H bending mode of adsorbed water molecules. In the low-frequency region below 1000 cm^−1^, distinct metal–oxygen stretching vibrations emerge at 620, 560, and 480 cm^−1^, corresponding to the Zn–O, Fe–O, and Cu–O bonds within the ZnO, ZnFe_2_O_4_, and CuO phases, respectively.

Meanwhile, the FTIR spectrum of exfoliated g-C_3_N_4_ displays a broad band at 3400–3200 cm^−1^ arising from the N–H stretching of residual amino groups and the O–H stretching of adsorbed water. The sharp band at 1630 cm^−1^ corresponds to the C=N stretching vibrations of the tri-*s*-triazine ring system. Intense bands in the 1570–1400 cm^−1^ region are attributed to aromatic C–N heterocycle stretching, while the band at 1320 cm^−1^ represents C–N stretching modes. The characteristic breathing vibration of the triazine ring appears at 810 cm^−1^, confirming the structural preservation of the heptazine units following exfoliation.

The spectrum of the GLTO composite integrates the features of both components, with specific peak shifts indicating interfacial bonding between the LTO and g-C_3_N_4_ phases. The broad O–H/N–H band at 3400–3200 cm^−1^ broadens further compared to the individual components, suggesting the formation of hydrogen bonds between the LTO surface hydroxyls and the g-C_3_N_4_ amino groups. The C=N stretching at 1630 cm^−1^ exhibits a subtle shift to a lower wavenumber, which may indicate electronic coupling through coordination between the nitrogen lone pairs of g-C_3_N_4_ and the unsaturated metal sites on the LTO surface. Furthermore, the aromatic C–N bands (1570–1400 cm^−1^) display an intensity redistribution, suggesting π-electron interactions with the oxide surface. The triazine ring bending at 810 cm^−1^ is retained, while the metal–oxygen vibrations at 620, 560, and 480 cm^−1^ show slight broadening. Collectively, these spectral changes suggest the presence of interfacial contact and potential electronic interactions between the two phases *via N*-metal coordination and hydrogen bonding networks.

Upon RhB adsorption, spectral modifications reflect the dye binding mechanism through multiple interaction sites (Fig. 2b). The O–H/N–H region broadens owing to hydrogen bonding between the dye's carboxyl and amine groups with both the LTO surface hydroxyls and the g-C_3_N_4_ amino groups. The C=N band shifts from 1630 to 1625 cm^−1^ with considerable broadening, suggesting π–π stacking interactions between the aromatic rings of the dye and the tri-*s*-triazine units of g-C_3_N_4_. Furthermore, new shoulders emerge at 1595 and 1460 cm^−1^, corresponding to adsorbed dye ring vibrations. Simultaneously, the metal–oxygen bands exhibit slight intensity attenuation without any positional shift. This behavior indicates an electrostatic attraction between the cationic RhB molecules and the negatively charged oxide surfaces, occurring without the disruption of the underlying coordination bonds.

Conversely, Indigo Carmine (IC) adsorption produces distinctly different spectral features characteristic of anionic dye interactions. The O–H/N–H region exhibits more pronounced broadening, indicating strong hydrogen bonding between the sulfonate and amine groups of the dye and the surface hydroxyls of the composite. The C=N band shifts further to 1620 cm^−1^, suggesting π–π interactions between the indole aromatic system of IC and the g-C_3_N_4_ surface. Notably, new bands emerge at 1120 and 1040 cm^−1^, corresponding to sulfonate S=O stretching, which provides clear evidence for IC uptake. The C–N stretching shifts to 1310 cm^−1^, reflecting contributions from the indole heterocycles. While the preserved metal–oxygen vibrations indicate structural integrity, subtle intensity variations suggest electrostatic interactions between the anionic sulfonate groups and the positively charged metal centers (Zn^2+^, Cu^2+^, Fe^3+^), potentially involving weak coordination through sulfonate-metal bonding. These distinct spectral fingerprints—enhanced hydrogen bonding and sulfonate bands for IC, compared to π–π interactions and carboxylate involvement for RhB—demonstrate the versatile adsorption capacity of the composite *via* complementary binding mechanisms.

The optical properties and band structures of ZnO, g-C_3_N_4_, LTO, and the GLTO composite were investigated using UV-Vis diffuse reflectance spectroscopy. The absorption spectra are shown in Fig. 3a, with the corresponding Tauc plots for direct band gap determination presented in Fig. 3b. Pristine ZnO exhibits a sharp absorption edge at approximately 380 nm, with negligible absorption beyond 400 nm. The Tauc plot yields a band gap of 3.2 eV, confirming its wide-band-gap semiconductor nature, which restricts its photoactivation to the UV region. Exfoliated g-C_3_N_4_ displays an absorption edge extending to approximately 450 nm, with a calculated band gap of 2.9 eV. This is characteristic of its visible-light response originating from the tri-*s*-triazine units, though the absorption tail remains limited beyond 500 nm.

**Fig. 3:**
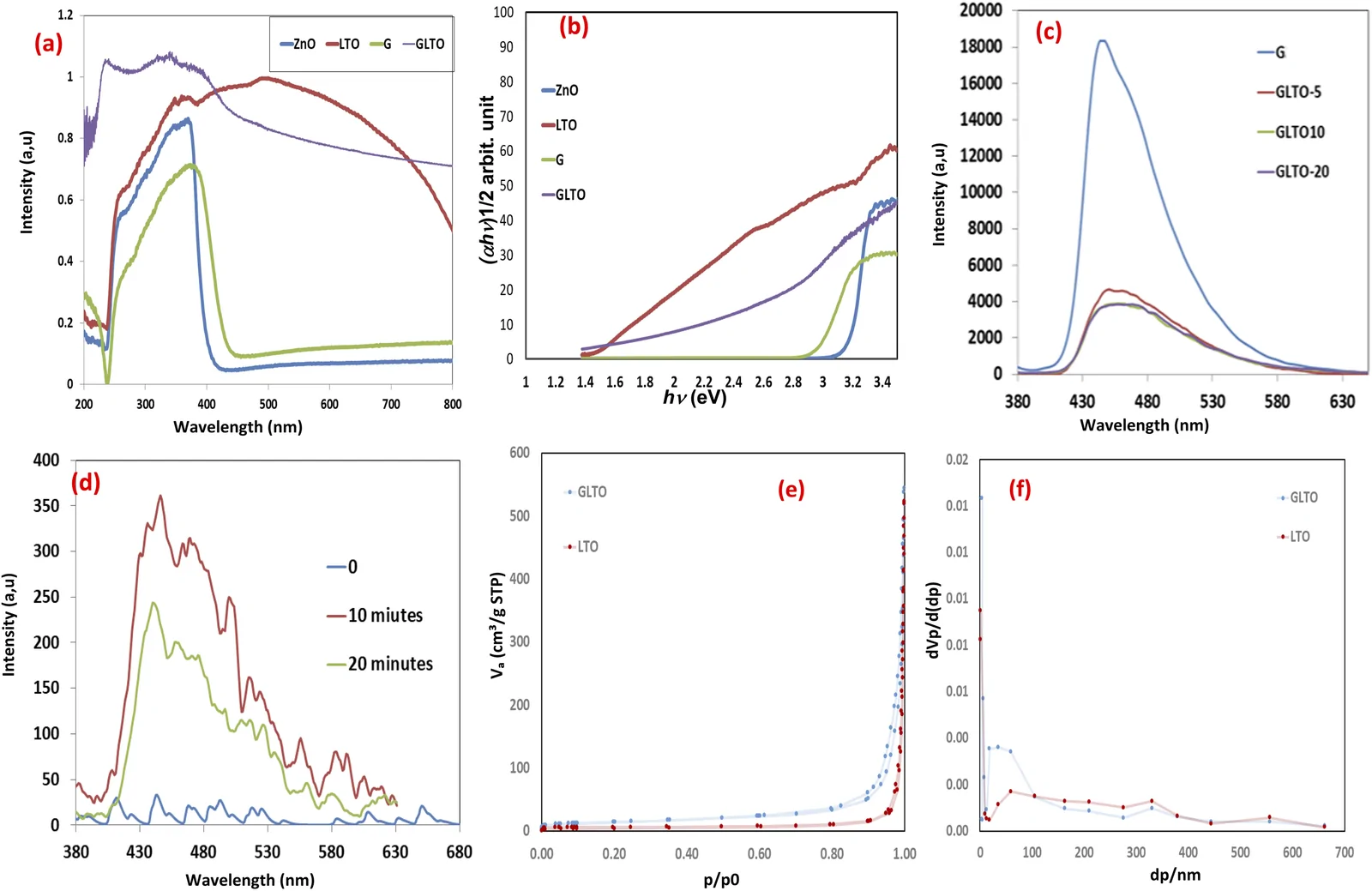
Characterization of the synthesized samples: ZnO, g-C_3_N_4_ (G), LTO, and GLTO. (a) UV-Vis DRS spectra, (b) Tauc plots, (c) PL spectra of g-C_3_N_4_ and GLTO with different ratios of LTO, (d) PL spectra of the terephthalic-acid probe experiment for ˙OH generation, (e) N_2_ adsorption–desorption isotherms, and (f) corresponding pore size distribution curves.

The LTO heterojunction exhibits different optical behavior, featuring a continuous absorption that spans the visible region (400–800 nm), with a tail extending beyond 700 nm. This red shift arises from the interaction among the mixed oxide phases generated during the LTH topotactic transformation. Within this ternary matrix, ZnFe_2_O_4_ typically absorbs visible light owing to its spinel structure, while CuO further extends the absorption through d–d transitions. The Tauc plot analysis reveals a narrowed band gap of approximately 1.5 eV for LTO, indicating that the LTH-derived mixed oxides can absorb visible light across a wide spectral range.

The GLTO composite integrates the absorption features of both components, showing enhanced absorption across the visible region. The absorption spectrum shows increased intensity across the visible region compared to bare g-C_3_N_4_, while maintaining the extended red response characteristic of LTO. The absorption edge exhibits a shift toward longer wavelengths, accompanied by continuous absorption through the visible range. Tauc plot analysis of the GLTO composite reveals a well-defined linear region yielding a band gap of approximately 2.4 eV. This intermediate value, positioned between the 2.9 eV of g-C_3_N_4_ and the 1.5 eV of LTO, is consistent with electronic interaction at the heterojunction interface. The reduction from 2.9 to 2.4 eV upon LTO integration suggests changes in the local electronic environment and may facilitate charge transfer between the components.

The charge carrier dynamics within the heterojunction were examined using photoluminescence (PL) spectroscopy. Pristine g-C_3_N_4_ exhibits an emission peak centered at approximately 460 nm, arising from the recombination of photogenerated electron–hole pairs, which can limit its photocatalytic efficiency. The integration of LTO reduces this emission (Fig. 3c). While the GLTO-5 composite shows a moderate reduction in PL intensity, higher loadings (GLTO-10 and GLTO-20) show a greater and comparable reduction, with approximately 80% lower PL intensity than bare g-C_3_N_4_. This quenching indicates reduced radiative recombination and is consistent with improved charge separation in the GLTO composite. Rather than being taken as direct proof of a specific charge-transfer mechanism, the observed PL quenching provides supporting evidence for improved charge separation and is consistent with the proposed S-scheme charge-transfer pathway, in which interfacial charge transfer may preserve the more reactive charge carriers.

Hydroxyl radical (˙OH) generation under visible-light irradiation was evaluated using terephthalic acid (TA) as a fluorescent probe. TA selectively reacts with ˙OH to form highly fluorescent 2-hydroxyterephthalic acid. Fig. 3d displays the time-dependent PL spectra of the TA solution containing the GLTO-20 composite under visible-light irradiation. The fluorescence intensity at 425 nm progressively increased over 0 to 50 min, indicating the generation of ˙OH during the illumination period under the tested conditions. The continuous rise in intensity without reaching an apparent plateau indicates continued radical generation under the tested conditions.

This ˙OH generation, inferred from the PL results, is consistent with the enhanced charge separation observed for GLTO. According to the proposed S-scheme pathway, the photogenerated holes are retained in the valence band of the LTO component, while the reducing electrons are retained in the conduction band of g-C_3_N_4_. The retained electrons can facilitate the reduction of Fe^3+^/Cu^2+^ species and thereby promote H_2_O_2_ activation through Fenton-like reactions, providing a plausible source of the observed ˙OH radicals. Meanwhile, the holes retained on LTO can contribute to the surface oxidation of adsorbed organic species. Thus, the increase in ˙OH-related TA fluorescence observed over 50 min is consistent with continued photo-Fenton activity and charge separation in the GLTO composite under the tested conditions.

The textural properties of the individual components and the GLTO composite were evaluated by N_2_ adsorption–desorption analysis. Pristine g-C_3_N_4_ exhibits a specific surface area of 12.24 m^2^ g^−1^ with an average pore diameter of 73.3 nm, while bare LTO shows a specific surface area of 15.1 m^2^ g^−1^ with an average pore diameter of 52.9 nm. Upon integration of the two components, the GLTO composite exhibits an approximately four-fold increase in specific surface area to 49.6 m^2^ g^−1^. This increase is accompanied by changes in the pore characteristics of the composite, indicating differences in the textural properties after heterojunction formation.

The N_2_ adsorption–desorption isotherms (Fig. 3e) exhibit Type IV behavior with H3 hysteresis loops for all samples, consistent with mesoporous structures containing slit-shaped pores. The GLTO composite displays a more pronounced hysteresis loop than the individual components, indicating a change in its mesoporous characteristics. The corresponding pore size distribution curves (Fig. 3f) show that GLTO has a distribution centered in the mesoporous region. In contrast, LTO shows a broader distribution extending toward larger pores, while g-C_3_N_4_ displays a wider pore size distribution. The total pore volume increases from 0.2243 cm^3^ g^−1^ for g-C_3_N_4_ and 0.2002 cm^3^ g^−1^ for LTO to 0.3845 cm^3^ g^−1^ for GLTO, while the average pore diameter decreases from 73.3 nm and 52.9 nm to 31.0 nm, respectively. These changes in surface area, pore volume, and pore diameter indicate the formation of a porous GLTO structure with a higher accessible surface area and a pore distribution centered in the mesoporous range.

The morphological features and interfacial structures of the prepared materials were examined using transmission electron microscopy (TEM) and high-resolution TEM (HRTEM) (Fig. 4a–f). Fig. 4a displays the exfoliated g-C_3_N_4_ nanosheets, which exhibit a characteristic wrinkled, lamellar morphology and a translucent appearance, confirming the successful exfoliation into few-layer sheets. The folded edges and overlapping regions indicate a flexible two-dimensional structure with a high aspect ratio.

**Fig. 4:**
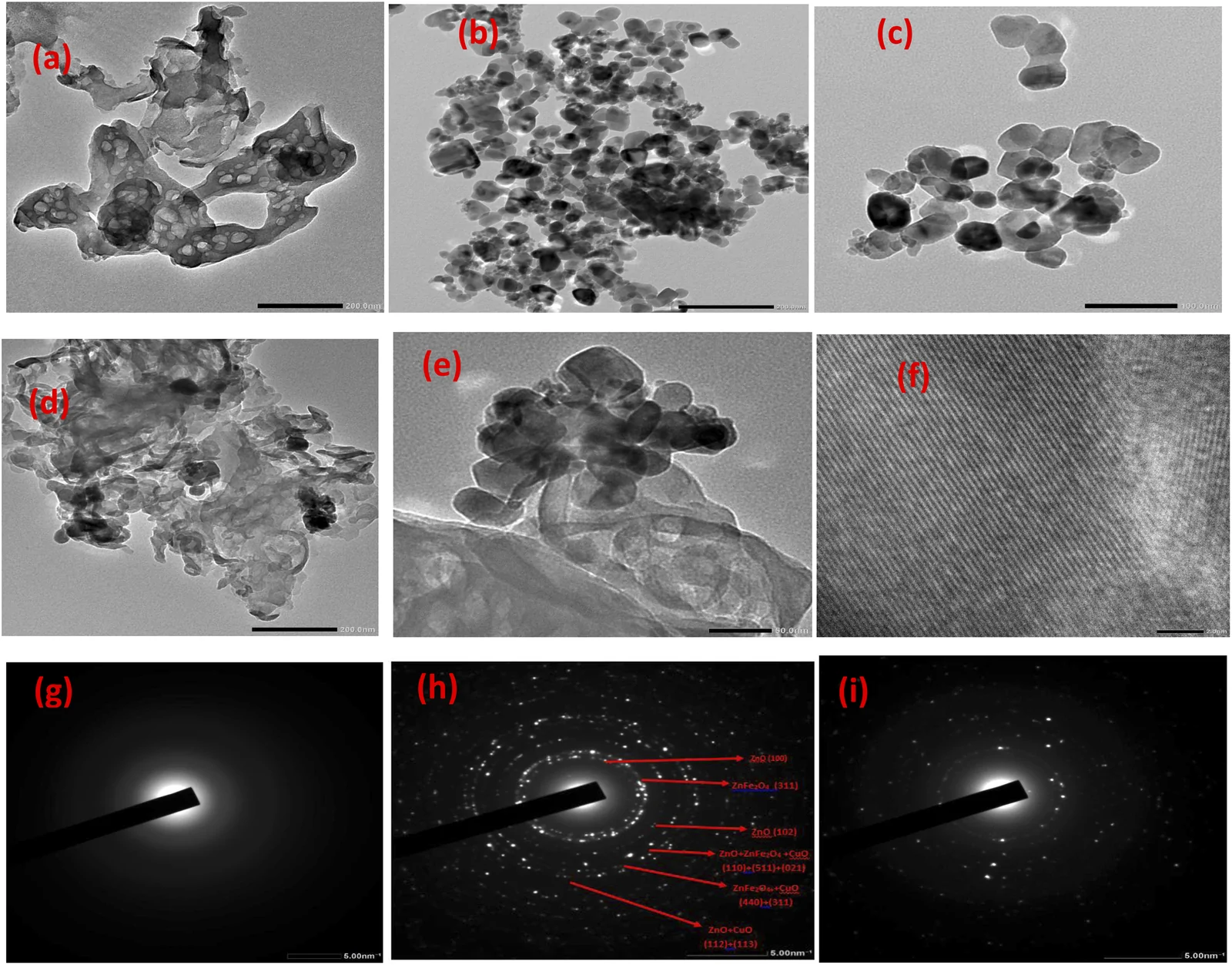
TEM images of (a) G, (b and c) LTO at different magnifications, (d and e) GLTO at different magnifications, (f) HRTEM image of GLTO, and SAED patterns of (g) G, (h) LTO, and (i) GLTO.

Fig. 4b and c reveal the LTO morphology at different magnifications. The ternary oxide forms irregular nanoparticles with dimensions ranging from 20 to 50 nm, exhibiting moderate aggregation due to the thermal transformation from the layered triple hydroxide precursor. HRTEM lattice fringe spacing measurements confirm the coexistence of the crystalline phases: *d*-spacings of 0.28 nm correspond to the ZnFe_2_O_4_ (220) planes, 0.26 nm matches the ZnO (002) planes, and 0.23 nm is consistent with the CuO (111) planes. The intimate contact between these crystalline domains at the nanoscale confirms the successful formation of the ternary heterojunction without macroscopic phase separation.

Fig. 4d and e display the morphology of the GLTO composite. The LTO nanoparticles are uniformly dispersed across the g-C_3_N_4_ nanosheet surface, with no evidence of severe aggregation. The intimate interfacial contact between the oxide nanoparticles and the carbon nitride sheets is apparent, with LTO particles appearing partially embedded within or securely anchored onto the wrinkled g-C_3_N_4_ surface. This close contact provides the extensive heterojunction interfaces essential for efficient charge transfer. The morphological evolution from the individual components to the composite is structurally significant. While bare g-C_3_N_4_ shows smooth sheet surfaces and LTO alone forms clustered nanoparticles, the integration in GLTO demonstrates a mutual dispersion effect. The g-C_3_N_4_ sheets effectively prevent LTO nanoparticle agglomeration, and the anchored LTO nanoparticles simultaneously hinder the restacking of the g-C_3_N_4_ sheets. This structural interplay, observed *via* TEM, directly correlates with the significant surface area enhancement determined by the BET analysis.

Furthermore, HRTEM analysis of the composite interface (Fig. 4f) shows the preserved lattice fringes of both components with clear interfacial boundaries. This observation indicates that the heterojunction forms through physical assembly while preserving the intrinsic crystalline structure of the respective phases, providing direct evidence of the interfacial contact between the two components.

The selected area electron diffraction (SAED) pattern of exfoliated g-C_3_N_4_ (Fig. 4g) exhibits two diffuse concentric rings with measured *d*-spacings of approximately 0.68 nm and 0.33 nm. These correspond to the (100) in-plane structural packing and the (002) interlayer stacking of the heptazine network, respectively. In contrast, the SAED pattern of bare LTO (Fig. 4h) displays sharp polycrystalline rings composed of discrete bright spots, indicating a high degree of crystallinity. Indexing of these rings confirms the coexistence of the three metal oxide phases: rings at 0.48 nm and 0.29 nm correspond to the ZnFe_2_O_4_ spinel (111) and (220) planes; rings at 0.28 nm, 0.26 nm, and 0.25 nm match the ZnO wurtzite (100), (002), and (101) planes; and additional rings at 0.25 nm and 0.23 nm are attributed to the CuO tenorite (002) and (111) planes. The sharp, spotty nature of these rings reflects the well-crystallized state of the nanoparticles following the thermal transformation of the LTH precursor.

The SAED pattern of the GLTO composite (Fig. 4i) is dominated by the sharp diffraction rings of LTO, whereas the characteristic diffuse rings of g-C_3_N_4_ become less distinct. The intense crystalline diffraction spots from the LTO nanoparticles effectively overshadow the weaker diffuse scattering of the carbon nitride support. This feature is consistent with the presence of LTO nanoparticles on the g-C_3_N_4_ sheets within the selected area. Nevertheless, faint diffuse rings remain discernible in the background, indicating the presence and structural preservation of g-C_3_N_4_ in the composite. The spatial overlap of these diffraction features within the same aperture area provides further crystallographic evidence of the coexistence of LTO and g-C_3_N_4_ within the selected region, supporting the formation of the heterojunction.

The surface morphology of the individual components and the resulting composite was examined using field-emission scanning electron microscopy (FESEM). Fig. 5a and b display the bare LTO at different magnifications. The ternary oxide forms irregular nanoparticle aggregates ranging in size from 30 to 80 nm. The surface appears rough with visible interparticle voids and particle clustering. These aggregates exhibit no preferential orientation, consistent with the morphology of the oxide particles following the thermal transformation. In contrast, Fig. 5c displays the exfoliated g-C_3_N_4_. The nanosheets exhibit a wrinkled morphology with folded edges and relatively smooth basal planes. Although the sheets stack loosely with visible interlayer gaps, the individual sheet surfaces remain relatively featureless.

**Fig. 5:**
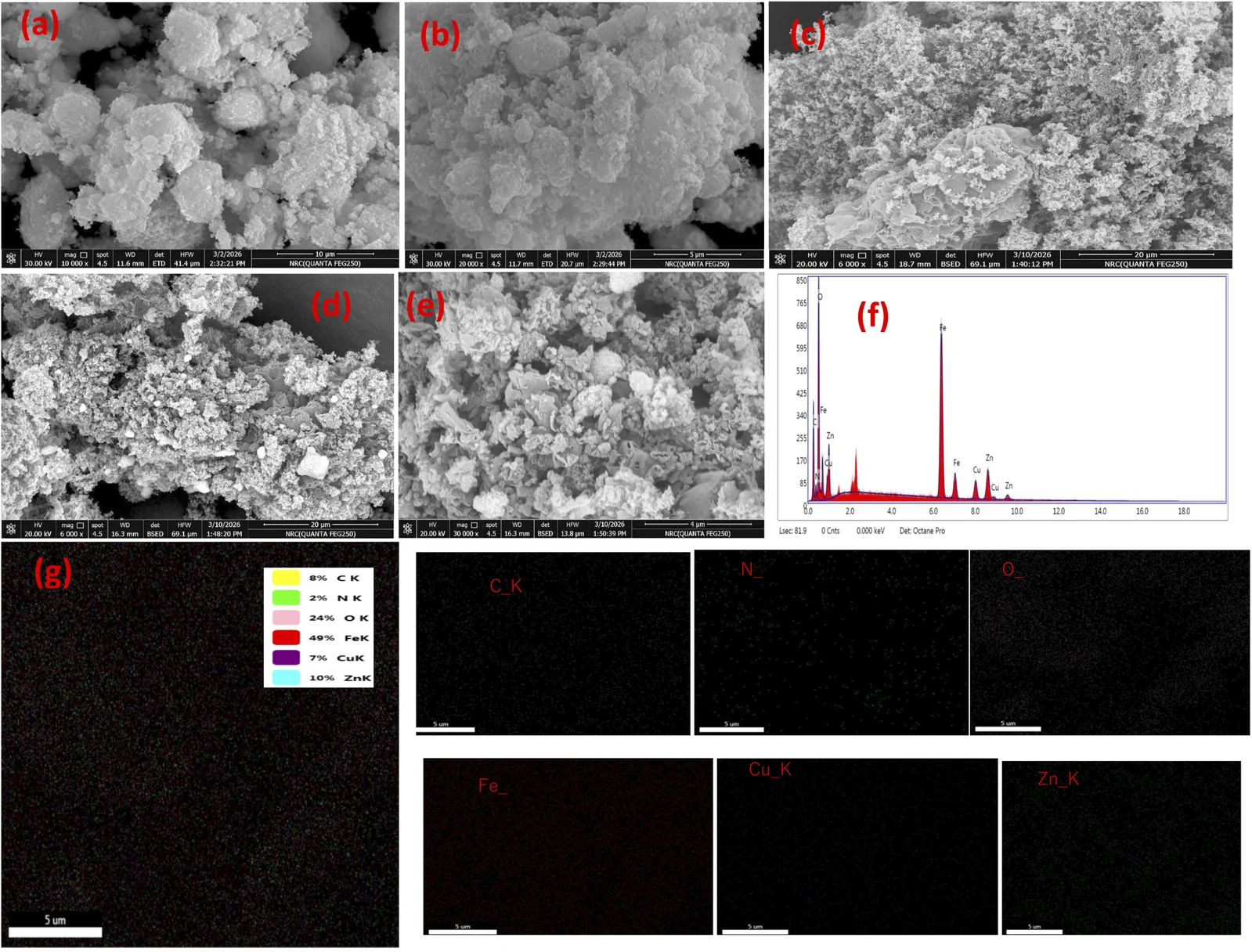
SEM images of (a and b) LTO at different magnifications, (c) g-C_3_N_4_ (G), (d and e) GLTO at different magnifications, (f) EDS spectrum of GLTO, and (g) elemental mapping of GLTO.

The morphological evolution following the heterojunction formation is evident in Fig. 5d and e. At a lower magnification (Fig. 5d), bright spots corresponding to the LTO nanoparticles are distributed across the darker g-C_3_N_4_ surface. This contrast arises from the higher electron density of the multimetal oxides compared to the carbon-based support. The distribution appears relatively homogeneous without large aggregates, indicating effective dispersion during the ultrasonic assembly. At a higher magnification (Fig. 5e), individual nanoparticles become clearly distinguishable, with sizes of 20–50 nm distributed on the wrinkled sheet surface. The nanoparticles appear partially embedded within, rather than merely resting upon, the sheets, indicating close interfacial contact. Furthermore, the characteristic wrinkled morphology of g-C_3_N_4_ remains visible beneath the nanoparticle layer, indicating that the assembly process preserves the morphology of the support. The surface coverage observed at this magnification indicates the distribution of LTO nanoparticles across the g-C_3_N_4_ sheets.

Fig. 5f presents the energy-dispersive X-ray spectroscopy (EDS) spectrum, confirming the presence of all constituent elements within the composite. Furthermore, elemental mapping images (Fig. 5g) reveal the spatial distribution of each element across the selected area. The carbon and nitrogen maps exhibit similar spatial patterns, consistent with the presence of the g-C_3_N_4_ matrix. The oxygen map correlates with the metal distributions, consistent with the oxide nature of the LTO phase. The iron, copper, and zinc maps display a similar spatial distribution across the selected area, indicating a relatively homogeneous distribution of the three transition metals without obvious localized segregation at the mapping scale. This elemental distribution, together with the interfacial contact observed in the FESEM and TEM analyses, supports the formation of a well-dispersed GLTO composite.

### 3.2 Adsorption studies

The adsorption of RhB onto the synthesized GLTO composite was examined as a function of contact time to elucidate the rate-controlling mechanisms. The adsorption profile exhibits distinct kinetic phases. During the initial 15 minutes, the uptake rises sharply to 5.66 mg g^−1^, representing 84% of the equilibrium capacity (6.73 mg g^−1^) achieved at 30 minutes. This rapid initial phase corresponds to rapid surface uptake, which may be related to the availability of surface binding sites, including the surface hydroxyl groups on the LTO nanoparticles and the exposed triazine units on the g-C_3_N_4_ sheets. The steep initial uptake is consistent with the mesoporous architecture (31.0 nm pore diameter), which may facilitate the diffusion of the dye toward the available adsorption sites.

To further elucidate the adsorption kinetics, the experimental data were fitted to the pseudo-first-order (PFO) and pseudo-second-order (PSO) kinetic models (Fig. 6a and b). Nonlinear regression fitting was performed using Origin software, and the corresponding kinetic parameters were obtained from the nonlinear fits. The PFO model yields a calculated equilibrium capacity of 7.29 mg g^−1^ with a rate constant (*k*_1_) of 0.095 min^−1^ and a correlation coefficient (*R*^2^) of 0.982. This calculated capacity deviates by only 8.3% from the experimental value of 6.73 mg g^−1^, indicating close agreement. Conversely, the PSO model produces a calculated capacity of 9.90 mg g^−1^, overestimating the experimental value by approximately 47%, with a rate constant (*k*_2_) of 0.008 g mg^−1^ min^−1^ and an *R*^2^ of 0.967 (Table 1). The lower agreement between the calculated and experimental equilibrium capacities, together with the lower *R*^2^ value, indicates that the PFO model provides a better description of the adsorption kinetics under the tested conditions. However, a better PFO fit alone should not be taken as proof of a physisorption mechanism. The observed adsorption behavior may involve several interactions, including hydrogen bonding between the carboxyl/amine groups of the dye and the surface hydroxyls, as well as π–π stacking interactions between the aromatic rings and the triazine units, as suggested by the FTIR analysis.

**Fig. 6:**
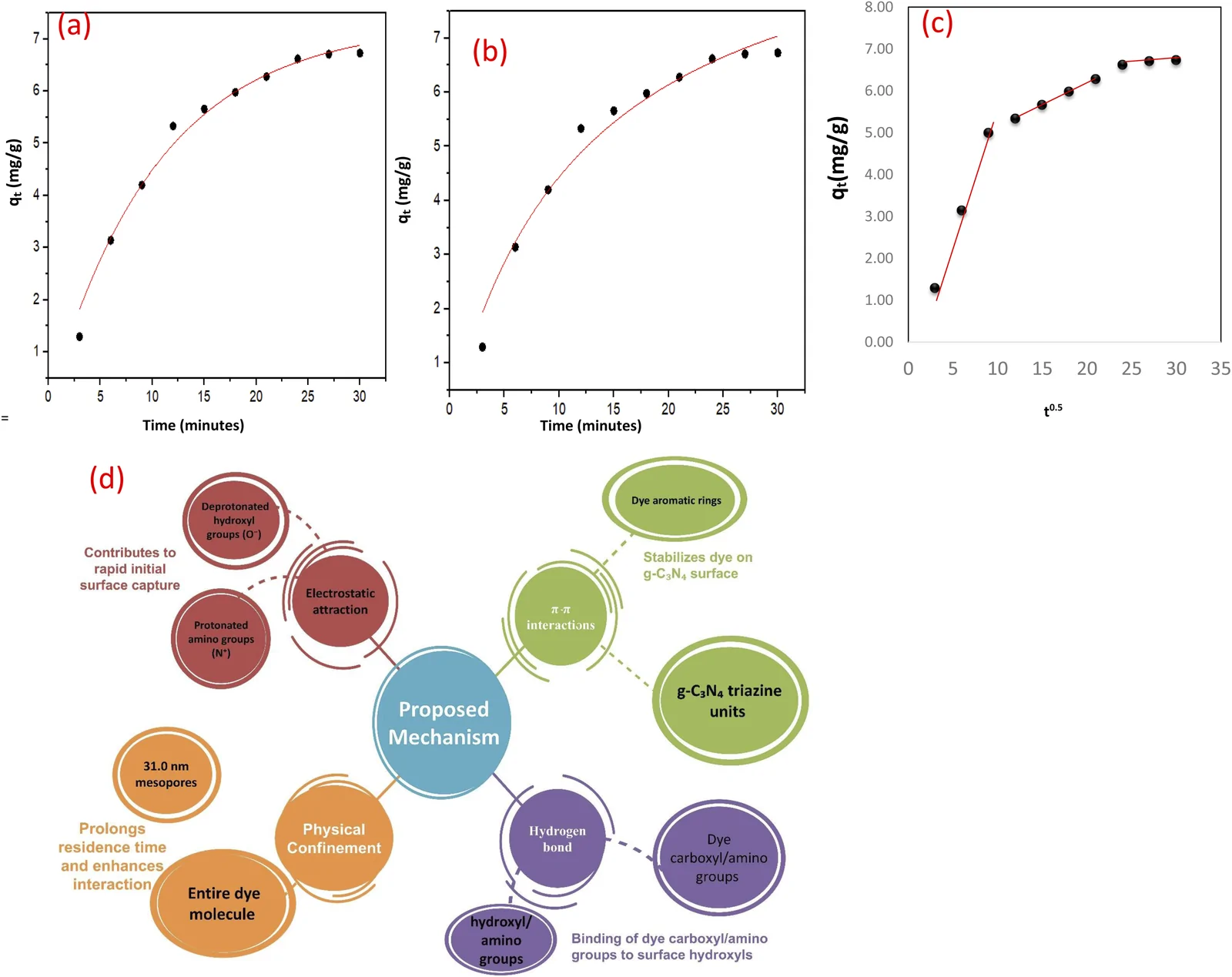
Kinetics of RhB dye adsorption onto GLTO: (a) nonlinear PFO kinetic model, (b) nonlinear PSO kinetic model, (c) intraparticle diffusion (IPD) model, and (d) schematic illustration of the proposed adsorption mechanisms of RhB onto GLTO.

**Table 1:** Kinetics model parameters

Model		
**Pseudo first order**		
*q*_exp_ (mg g^−1^)		6.73
*q*_Calc_ (mg g^−1^)		7.29
*K*_1_ (min^−1^)		0.095
*R* ^2^		0.982

**Pseudo second order**		
*q*_exp_ (mg g^−1^)		6.73
*q*_Calc_ (mg g^−1^)		9.9
*K*_2_ (g mg^−1^ min^−1^)		0.008
*R* ^2^		0.967

**Weber- Morris**		
First stage	*K* (mg g^−1^ min^−0.5^)	0.617
	*C*	0.564
Second stage	*K* (mg g^−1^ min^−0.5^)	0.105
	*C*	4.07
Third stage	*K* (mg g^−1^ min^−0.5^)	0.018
	*C*	6.1

#### 3.2.1 Intraparticle diffusion model

The intraparticle diffusion model developed by Weber and Morris was applied to further investigate the rate-controlling steps (Fig. 6c). The plot of *q*_*t*_*versus t*^0.5^ reveals three distinct linear segments, indicating that multiple diffusion processes contribute to the adsorption process. The first stage exhibits the highest diffusion rate constant of 0.617 mg g^−1^ min^−0^·^5^ with an intercept of 0.564. This stage corresponds to rapid adsorption at the external surface and boundary layer diffusion, where dye molecules migrate from the bulk solution toward the external surface of the GLTO composite. The non-zero intercept indicates a contribution from boundary layer diffusion, consistent with the initial rapid uptake.

The second stage shows a reduced rate constant of 0.105 mg g^−1^ min^−0^·^5^ with an increased intercept of 4.07. This stage represents a slower diffusion process within the porous structure, where dye molecules progressively access the available adsorption sites. The decreased rate may reflect increased diffusion resistance within the pore structure, while the higher intercept indicates that intraparticle diffusion contributes after the initial external adsorption stage. The third stage exhibits the lowest rate constant of 0.018 mg g^−1^ min^−0^·^5^ with an intercept of 6.10, approaching the equilibrium capacity. This final stage corresponds to a slower adsorption process as the system approaches equilibrium. The low slope indicates a reduced diffusion rate during this stage.

None of the three linear segments passes through the origin, indicating that intraparticle diffusion is not the sole rate-controlling step. The multilinear nature of the plot indicates that both boundary layer and intraparticle diffusion contribute to the overall adsorption kinetics, with their relative contributions varying across the different stages. The decreasing rate constants from stage one to stage three indicate changes in the diffusion rate as adsorption proceeds from the bulk solution to the external surface and subsequently within the porous structure. This multistep diffusion behavior is consistent with the porous structure of GLTO, where the 31.0 nm average pore diameter may facilitate dye diffusion while the subsequent diffusion within the porous structure may involve additional resistance.

### 3.3 Photo-Fenton catalytic performance

To establish the baseline performance under different operational conditions, control experiments were conducted (Fig. 7a and b). Under direct photolysis (visible light + H_2_O_2_ without any catalyst), RhB removal was negligible, indicating that direct photolysis contributes little to RhB degradation under the tested conditions. Under dark Fenton conditions (GLTO composite + H_2_O_2_ in the dark), only a minor decrease in dye concentration was observed, which rapidly reached an early plateau. This limited dark activity indicates that the Fenton-like reaction proceeds to a limited extent in the absence of light, resulting in limited degradation of RhB under the tested conditions.

**Fig. 7:**
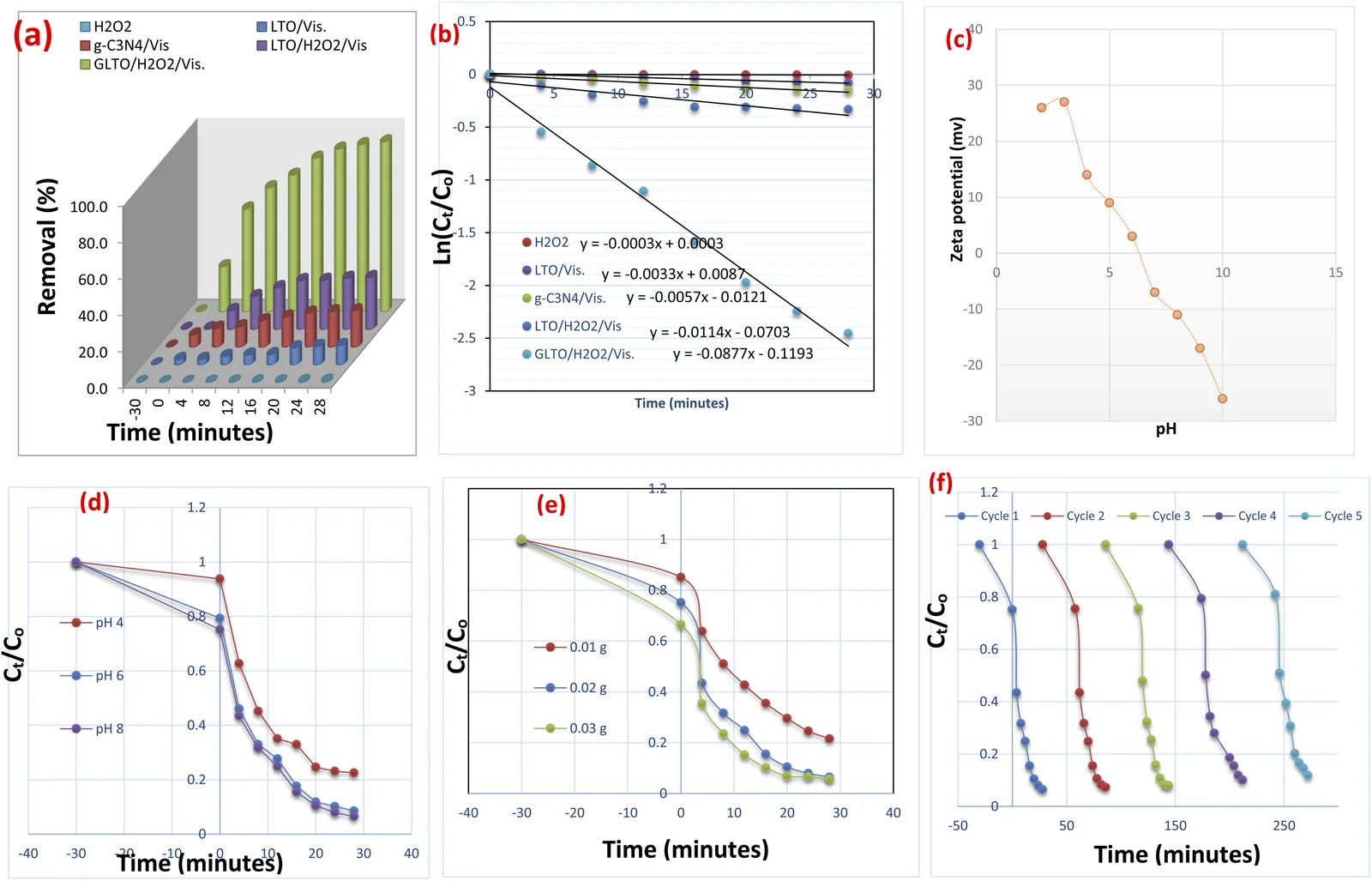
Evaluation of the photo-Fenton process for RhB removal using GLTO: (a) time-dependent degradation profiles, (b) PFO kinetic modeling with comparison of apparent rate constants (k), (c) zeta potential of GLTO, (d) effect of initial pH, (e) effect of catalyst (GLTO) dose, and (f) regeneration performance over five cycles.

When evaluating the standalone components under visible-light irradiation, bare LTO exhibits limited activity, achieving only 10.6% degradation after 28 minutes despite its narrow band gap of 1.5 eV. This performance may be related to the aggregated morphology of the bare LTO nanoparticles, which limits accessible surface-active sites and restricts dye-catalyst contact. Additionally, even when charge separation occurs, the photogenerated charge carriers may undergo radiative recombination before participating in surface redox reactions, consistent with the PL intensity observed for bare LTO. Similarly, pure g-C_3_N_4_ demonstrates slightly higher but still limited performance, reaching 20% degradation. Although g-C_3_N_4_ absorbs visible light through its tri-*s*-triazine units, its PL emission indicates that a substantial fraction of the photogenerated electron–hole pairs recombine before participating in surface reactions, leaving fewer carriers available for these reactions.

To investigate the effect of combining the transition metal oxide with an oxidant, the LTO/H_2_O_2_ system was evaluated under visible light, reaching 28.6% degradation. This represents a 2.7-fold enhancement over light-driven LTO alone, as the addition of H_2_O_2_ provides an external electron acceptor that captures photogenerated electrons, partially suppressing recombination and generating hydroxyl radicals through reduction reactions. Meanwhile, the Fe^3+^/Fe^2+^ and Cu^2+^/Cu^+^ redox couples within the matrix participate in Fenton-like chemistry. However, the degradation profile plateaus after 16 minutes at 28.6%, indicating limited further degradation under the tested conditions. This plateau may be related to the aggregated morphology of bare LTO, where electron transfer to H_2_O_2_ may occur mainly at accessible outer surface sites, while the cationic RhB molecules may experience electrostatic repulsion from the positively charged oxide surface at pH levels below its point of zero charge (pH_p_zc).

The formulation of the pure photocatalytic composite system (GLTO composite + visible light without H_2_O_2_) showed improved but still limited degradation efficiency. This indicates that while the integrated heterojunction generates charge carriers under illumination, the absence of an efficient external electron acceptor may lead to electron accumulation and limit the overall reaction rate.

In contrast, the complete photo-Fenton system (GLTO + H_2_O_2_ + visible light) improves the reaction kinetics, achieving 93.5% RhB degradation efficiency with sustained degradation throughout the 28 minutes reaction period without any apparent plateau. This enhancement may arise from the combined structural and electronic characteristics of the composite. First, the g-C_3_N_4_ sheets limit LTO nanoparticle agglomeration, increasing the accessible specific surface area from 15.1 to 49.6 m^2^ g^−1^. Second, the formation of an S-scheme heterojunction facilitates spatial charge separation, as indicated by the approximately 80% PL quenching. Third, the proposed S-scheme pathway preserves reducing electrons on the g-C_3_N_4_ side, which can promote Fe^3+^/Fe^2+^ and Cu^2+^/Cu^+^ cycling and O_2_ activation, while oxidizing holes are retained on the LTO side. Finally, the hierarchical mesoporous structure (mean pore diameter, 31.0 nm) may facilitate the access of RhB and H_2_O_2_ to reactive surface sites. These effects collectively contribute to the observed photo-Fenton degradation performance.

#### 3.3.1 Surface-charge analysis and effect of initial pH

The surface charge characteristics of the GLTO composite were evaluated by zeta potential measurements across a pH range of 2 to 10, as shown in Fig. 7c. Under highly acidic conditions (pH 2 and 3), the zeta potential exhibits positive values of +26 mV and +27 mV, respectively. This positive surface charge may be associated with the protonation of surface hydroxyl groups (M-OH_2_^+^) on the LTO nanoparticles and amino groups (–NH_2_^+^) on the g-C_3_N_4_ sheets. As the pH increases, the positive charge gradually diminishes: +14 mV at pH 4, +9 mV at pH 5, and +3 mV at pH 6. The point of zero charge (pH_p_zc) is reached at approximately pH 6.5, where the net surface charge approaches zero. Beyond this point, the surface acquires a progressively negative charge: −7 mV at pH 7, −11 mV at pH 8, −17 mV at pH 9, and −26 mV at pH 10. This transition may be associated with the deprotonation of surface hydroxyl groups (M–O^−^) on LTO and nitrogen-containing surface groups on g-C_3_N_4_. The monotonic decrease in zeta potential with increasing pH is consistent with the amphoteric surface behavior of the GLTO composite, arising from the coexistence of the transition metal oxides (ZnFe_2_O_4_, ZnO, CuO) and the graphitic carbon nitride matrix. The relatively high absolute zeta potential values at the pH extremes indicate greater electrostatic repulsion between particles, while the pH_p_zc of 6.5 reflects the combined surface characteristics of the composite.

Furthermore, the influence of the initial solution pH on the photo-Fenton degradation of RhB over the GLTO composite was examined at pH 4, 6, and 8. During the dark adsorption equilibrium stage, the dye uptake increases progressively with pH, consistent with the surface charge characteristics of the catalyst. At pH 8 (above the pH_p_zc of 6.5), the negatively charged GLTO surface can electrostatically attract the cationic RhB molecules, thereby increasing pre-adsorption. Conversely, at pH 4 (below the pH_p_zc), the positively charged surface may electrostatically repel the cationic dye molecules, resulting in lower adsorption, whereas pH 6 exhibits an intermediate adsorption behavior near the point of zero charge.

Upon visible-light irradiation, the degradation efficiency follows the same pH-dependent trend observed for adsorption, with pH 8 showing higher degradation than pH 6, followed by pH 4 (Fig. 7d). The higher catalytic performance at pH 8 may be related primarily to electrostatic interactions; the increased pre-adsorption brings a higher concentration of dye molecules into contact with the surface-bound radical-generating sites. Furthermore, unlike homogeneous Fenton systems that can suffer from iron precipitation and deactivation at alkaline pH, the heterogeneous nature of the GLTO solid matrix may help maintain the availability of the Fe and Cu centers for redox cycling under the tested conditions. At pH 4, the electrostatic repulsion between the positively charged surface and the cationic dye may reduce the probability of localized ˙OH attacks. Additionally, the higher proton concentration may contribute to hydroxyl radical scavenging, thereby slightly lowering the overall degradation efficiency. The evaluated pH values exhibit degradation throughout the 28 minutes reaction period without an apparent plateau, indicating that the GLTO system remains active across the tested pH range (4–8). These results demonstrate that the GLTO composite maintains photo-Fenton activity over the investigated pH range, although the degradation efficiency depends on the solution pH and the associated surface charge characteristics.

#### 3.3.2 Effect of catalyst dose

The influence of GLTO loading on RhB degradation reveals a nonlinear relationship between catalyst dosage and photo-Fenton catalytic efficiency (Fig. 7e). During the dark adsorption phase, dye removal increases progressively with catalyst mass, indicating that a higher catalyst dosage provides additional surface area and available active sites for dye capture through hydrogen bonding, π–π interactions, and physical confinement.

Upon visible-light irradiation, all catalyst loadings achieve rapid degradation but exhibit distinctly different kinetic profiles. At the lowest dose, degradation proceeds steadily but remains incomplete, indicating that the limited availability of catalyst surface-active sites constrains radical generation. Increasing the dose to 0.02 g accelerates the reaction, achieving near-complete degradation. This enhancement may reflect the greater availability of catalyst surface area and the increased number of active sites, while the Fe^3+^/Fe^2+^ and Cu^2+^/Cu^+^ redox cycles contribute to the photo-Fenton process under the tested conditions.

A further increase to double the catalyst mass yields only a marginal improvement in the degradation rate. This plateau may arise from competing effects at higher catalyst loadings: increased particle concentration can cause light scattering and attenuation, thereby reducing photon penetration through the reaction medium, while particle aggregation may also reduce the accessibility of active sites. Although the dark adsorption results indicate that more catalyst surface is available at higher doses, the limited improvement in degradation during irradiation suggests that light availability may become an important factor at higher catalyst loadings. Therefore, the optimal dose represents a balance between the available catalyst surface and light penetration under the tested conditions. Below this dose, the available surface area may limit the reaction rate, whereas above it, reduced light penetration may limit the benefit of further increasing the catalyst loading. At the optimal loading, sufficient catalyst surface is available while light can still penetrate the suspension, resulting in high photo-Fenton degradation efficiency with the tested catalyst dose.

#### 3.3.3 Regeneration and reusability

The recyclability of the GLTO composite was evaluated through five consecutive photo-Fenton degradation cycles of RhB under identical experimental conditions (Fig. 7f). Following each cycle, the catalyst was recovered by centrifugation, thoroughly washed with deionized water and absolute ethanol, and dried prior to reuse. The GLTO composite maintained approximately 95% of its initial degradation efficiency after the fifth cycle, with a decrease of less than 5% over the five consecutive cycles. These results demonstrate that the GLTO composite retained most of its photocatalytic activity during repeated use under the tested conditions. The slight decrease in performance may be associated with catalyst loss during the repeated recovery procedure and/or partial accumulation of degradation intermediates on the catalyst surface.

#### 3.3.4 Performance in complex matrices

##### 3.3.4.1 Effect of H_2_O_2_ concentration

The role of H_2_O_2_ concentration in the GLTO photo-Fenton system was investigated by varying the initial H_2_O_2_ dosage from 0 to 5 mM under otherwise identical conditions (Fig. 8a). In the absence of H_2_O_2_, only 10.6% degradation of RhB is achieved after 28 minutes of visible-light irradiation, confirming that the photocatalyst alone cannot generate a sufficient quantity of ˙OH radicals without an external oxidant. Introducing H_2_O_2_ enhances the degradation efficiency. At a 1 mM H_2_O_2_ dosage, the degradation reaches approximately 78%, while increasing the concentration to 3 mM further improves the performance to an optimal 93.5% with a linear kinetic profile. However, raising the H_2_O_2_ concentration to 5 mM results in a slight decline in degradation efficiency (approximately 88%), indicating a critical threshold for the oxidant dosage.

**Fig. 8:**
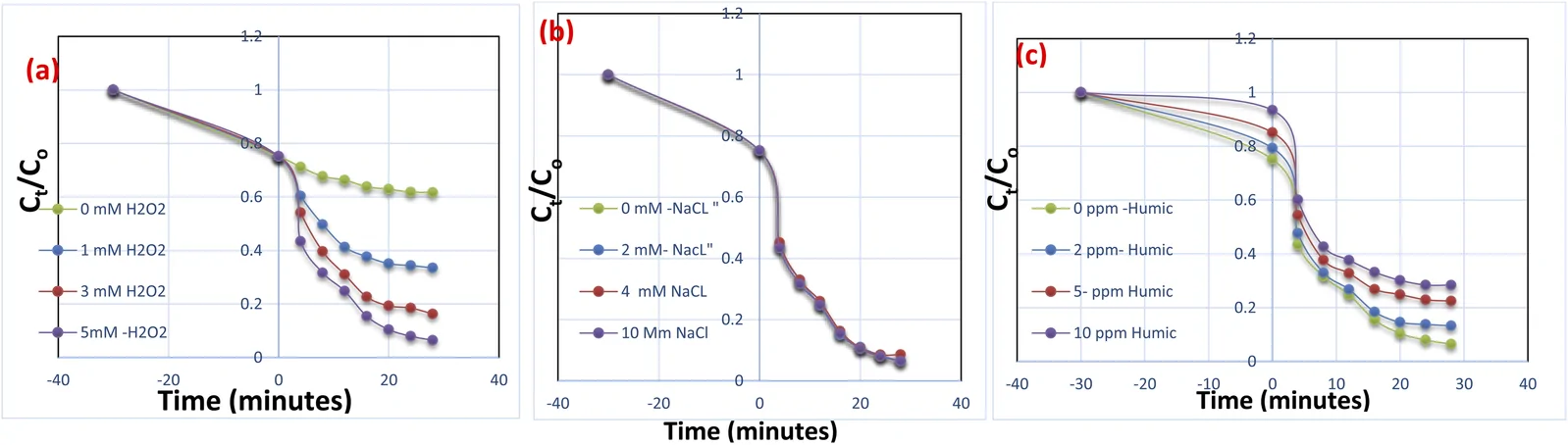
Photo-Fenton mineralization of RhB under visible light irradiation using GLTO as catalyst: control experiments showing the effect of (a) varying H_2_O_2_ concentration (including 0 mM H_2_O_2_ as a control without H_2_O_2_), (b) varying NaCl concentration, and (c) varying humic acid concentration on the degradation efficiency.

The enhancement in catalytic activity with increasing H_2_O_2_ concentration is attributed to the availability of H_2_O_2_ for reaction with surface Fe^2+^ and Cu^+^ species, thereby increasing ˙OH production through Fenton-like reactions. In addition, within the proposed S-scheme mechanism, the strongly reducing electrons retained on the g-C_3_N_4_ reduction side can promote O_2_ activation and facilitate the reduction of Fe^3+^/Cu^2+^ centers, thereby sustaining the redox cycles responsible for H_2_O_2_ activation.

Conversely, the observed efficiency decrease at 5 mM H_2_O_2_ can be attributed to the scavenging effect of excess H_2_O_2_. Excess H_2_O_2_ reacts with the highly reactive ˙OH to form the less reactive hydroperoxyl radicals (˙HO_2_) and can also consume photogenerated holes, thereby reducing the oxidation capacity of the system. Consequently, 3 mM H_2_O_2_ was selected as the optimal concentration for the GLTO/Vis system, providing a balance between radical generations and scavenging reactions.

##### 3.3.4.2 Performance in complex water matrices

The influence of salinity on the photo-Fenton degradation of RhB over the GLTO composite was examined by introducing NaCl at concentrations of 0, 2, 4, and 10 mM. Under dark conditions, the presence of NaCl does not significantly alter the initial adsorption capacity of the catalyst (Fig. 8b). Upon visible-light irradiation, the degradation kinetics at 2 mM NaCl closely match those observed in the absence of salt, indicating that low chloride levels do not significantly interfere with the radical generation pathway. At higher NaCl concentrations (4 and 10 mM), a slight retardation in degradation is observed, as reflected by marginally higher residual dye concentrations after the 28 minutes reaction period. This minor inhibition is attributed to the scavenging effect of chloride ions, which can react with highly reactive hydroxyl radicals to form less reactive chlorine-containing radical species. Even at the highest tested concentration (10 mM NaCl), the GLTO catalyst achieves approximately 90% degradation of RhB under the tested conditions. These results indicate that the GLTO composite maintains its photocatalytic performance in the tested NaCl-containing model water matrix.

Similarly, the presence of humic acid (HA), a ubiquitous natural organic matter, progressively suppresses the photo-Fenton degradation of RhB. During the dark equilibrium stage, higher HA concentrations increasingly reduce dye adsorption, indicating the competitive occupation of surface active sites by the HA macromolecules (Fig. 8c). Under visible-light irradiation, the degradation efficiency declines steadily with rising HA levels, dropping from 93.5% in the HA-free solution to approximately 87% at 2 mg L^−1^, 78% at 5 mg L^−1^, and 72% at 10 mg L^−1^. This inhibition may arise from a combination of three factors: HA acts as a competitive scavenger for hydroxyl radicals, absorbs a portion of the incident visible light (the inner filter effect), and physically blocks the active catalytic sites on the heterojunction surface. Despite these interferences, the GLTO composite retains over 70% efficiency even at a HA concentration of 10 mg L^−1^ under the tested conditions. This behavior may be related to the continued generation of reactive species through the GLTO heterojunction, which can partially compensate for the loss of reactive species in the presence of HA.

#### 3.3.5 Proposed photocatalytic degradation mechanism

Electrochemical impedance spectroscopy (EIS) and electron spin resonance (ESR) measurements were conducted to investigate interfacial charge transfer and the involvement of reactive species in the GLTO heterojunction (Fig. 9a and b). The EIS results show a decrease in charge-transfer resistance from approximately 1625 Ω cm^2^ for pristine g-C_3_N_4_ to 533 Ω cm^2^ for GLTO, which is consistent with lower resistance to interfacial charge migration. ESR measurements showed higher signal intensities for GLTO than for bare LTO. Bare LTO exhibited *g*-factors of 1.99213 and 2.05804, with a resonance field (*H*_0_) of 3473.3 G and a linewidth (Δ*H*) of 205.5 G, whereas GLTO exhibited *g*-factors of 2.0037 and 2.0548, *H*_0_ = 3453.9 G, and Δ*H* = 168.6 G. The observed *g*-factor values are consistent with paramagnetic species. Within the proposed S-scheme configuration, the electrons retained on the g-C_3_N_4_ side may participate in O_2_ reduction and Fe^3+^/Cu^2+^ redox cycling, while the holes retained on the LTO side may contribute to the oxidation of adsorbed organic species. The ESR results, together with the scavenger experiments, support the involvement of reactive species in the photocatalytic process. The scavenger experiments further indicate that ˙OH is the dominant reactive species under the tested conditions (Fig. 9c and d).

**Fig. 9:**
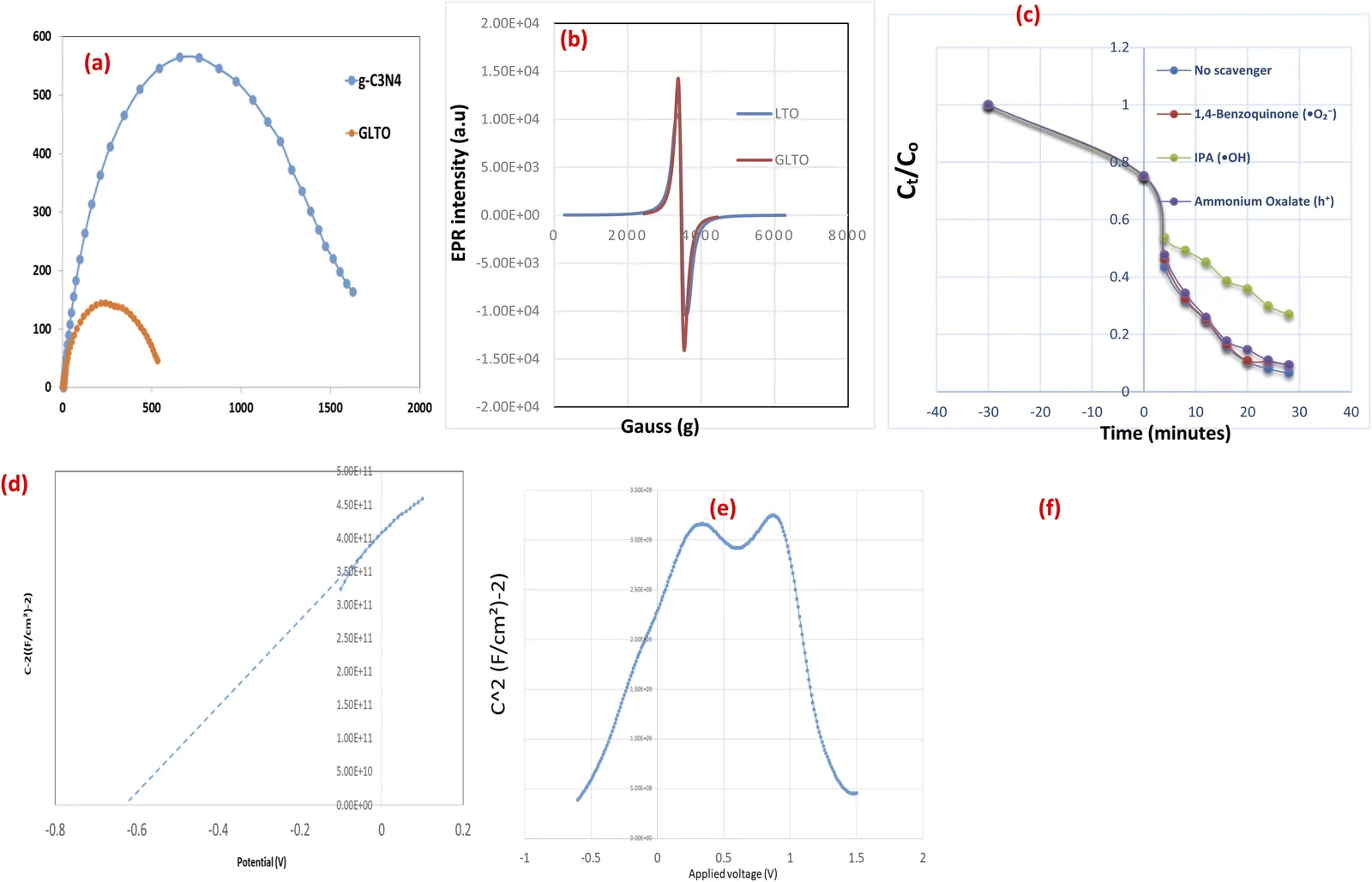
(a) EIS Nyquist plots of g-C_3_N_4_ and GLTO, (b) ESR spectra of LTO and GLTO, (c and d) effect of scavengers, and Mott–Schottky plots for (e) g-C_3_N_4_ and (f) LTO.

To understand the GLTO interface, the charge behavior of the standalone LTO system derived from the Zn–Cu–Fe layered triple hydroxide should first be considered. The LTO matrix contains n-type ZnO and ZnFe_2_O_4_ coupled with p-type CuO, providing the basis for internal p–n junctions and interfacial electric fields. The separate positive- and negative-slope regions observed in the Mott–Schottky response are consistent with these n-type and p-type contributions, respectively. The resulting charge separation can facilitate Fe^3+^/Fe^2+^ and Cu^2+^/Cu^+^ redox cycling and contribute to the photo-Fenton process. Nevertheless, standalone LTO shows limited photo-Fenton performance (28.6% degradation under the tested conditions). This limitation may be related to nanoparticle aggregation, restricted accessibility of surface-active sites, and electrostatic interactions with cationic RhB. These factors can limit the contribution of charge separation to the overall photocatalytic performance of bare LTO.

When the LTO multimetal oxide was integrated with exfoliated g-C_3_N_4_ nanosheets, an interfacial heterojunction was formed. The Mott–Schottky measurements were performed under the experimental conditions described in the SI. For g-C_3_N_4_, the positive-slope linear region gave a flat-band potential of −0.78 V *vs.* Ag/AgCl. For the n-type semiconductor, the CB position was estimated to be 0.10 V more negative than the flat-band potential, giving −0.88 V *vs.* Ag/AgCl (Fig. 9e). After conversion to the NHE scale using *E*_NHE = *E*_Ag/AgCl + 0.197 V, the CB position was estimated as −0.683 V *vs.* NHE. With an optical band gap of 2.90 eV obtained from the UV-Vis diffuse-reflectance measurement, the corresponding VB position was estimated as +2.217 V *vs.* NHE.

In contrast, the LTO composite exhibited positive- and negative-slope regions in its Mott–Schottky response. These regions were fitted separately to account for the n-type and p-type contributions of the multiphase LTO. The positive-slope region from −0.50 to 0.00 V *vs.* Ag/AgCl was fitted as the n-type region, giving an *x*-intercept of −0.652 ± 0.006 V *vs.* Ag/AgCl (*R*^2^ = 0.998). The negative-slope region from 1.00 to 1.20 V *vs.* Ag/AgCl gave an *x*-intercept of +1.332 ± 0.024 V *vs.* Ag/AgCl (*R*^2^ = 0.998). These results indicate the coexistence of n-type and p-type contributions in LTO, consistent with the ZnO/ZnFe_2_O_4_–CuO multiphase composition, rather than a single n-type semiconductor (Fig. 9f).

For the n-type contribution, the conduction-band position was estimated to be 0.10 V more negative than the flat-band potential. Thus, the n-type flat-band potential of −0.652 V *vs.* Ag/AgCl corresponds to an estimated CB potential of −0.752 ± 0.006 V *vs.* Ag/AgCl. Using the conversion equation *E*_NHE_ = *E*_Ag/AgCl_ + 0.197 V, the corresponding CB position is −0.555 ± 0.006 V *vs.* NHE. The optical band gap of LTO obtained from the UV-Vis diffuse-reflectance measurement was 1.50 eV, and the corresponding effective VB position was estimated using *E*_VB_ = *E*_CB_ + *E*_g_, giving +0.945 V *vs.* NHE. The negative-slope branch was used to identify the p-type contribution and was not combined with the n-type branch to define the optical band gap of LTO.

Accordingly, the band positions obtained from the Mott–Schottky and optical measurements are treated as estimated effective values. The uncertainty of the Mott–Schottky-derived flat-band and CB positions reflects the regression uncertainty of the respective linear fits, whereas the uncertainty of the VB position additionally depends on the uncertainty of the optical band-gap determination. The estimated band positions provide the basis for discussing the charge-transfer behavior of the GLTO interface, while the proposed interfacial band bending and charge redistribution are considered mechanistic interpretations rather than direct measurements of Fermi-level equilibration.

Under visible-light irradiation, the proposed S-scheme pathway favors the recombination of electrons from the CB of LTO with holes from the VB of g-C_3_N_4_. This process leaves the more reducing electrons on g-C_3_N_4_ and the oxidizing holes on the LTO side. The PL quenching, reduced EIS resistance, ESR, and scavenger experiments collectively support enhanced charge separation and reactive oxygen species generation, consistent with the proposed S-scheme pathway, although these results do not independently distinguish the S-scheme from all alternative charge-transfer mechanisms.

The retained g-C_3_N_4_ electrons can reduce O_2_ to ˙O_2_^−^ and participate in the regeneration of Fe^2+^ and Cu^+^ from Fe^3+^ and Cu^2+^, respectively, thereby supporting H_2_O_2_ activation and Fenton-like ˙OH generation. The holes retained on the LTO side can contribute to the oxidation of adsorbed organic species. Because the estimated effective VB of LTO is not sufficiently positive for direct water oxidation to ˙OH, ˙OH generation is attributed primarily to the Fe/Cu-mediated Fenton-like reactions rather than direct water oxidation. Together with the porous architecture (49.6 m^2^ g^−1^; mean pore diameter, 31.0 nm) and interfacial contact, these processes are consistent with the observed RhB removal of 93.5% within 28 min and TOC reduction of 65% (Fig. 10).

**Fig. 10:**
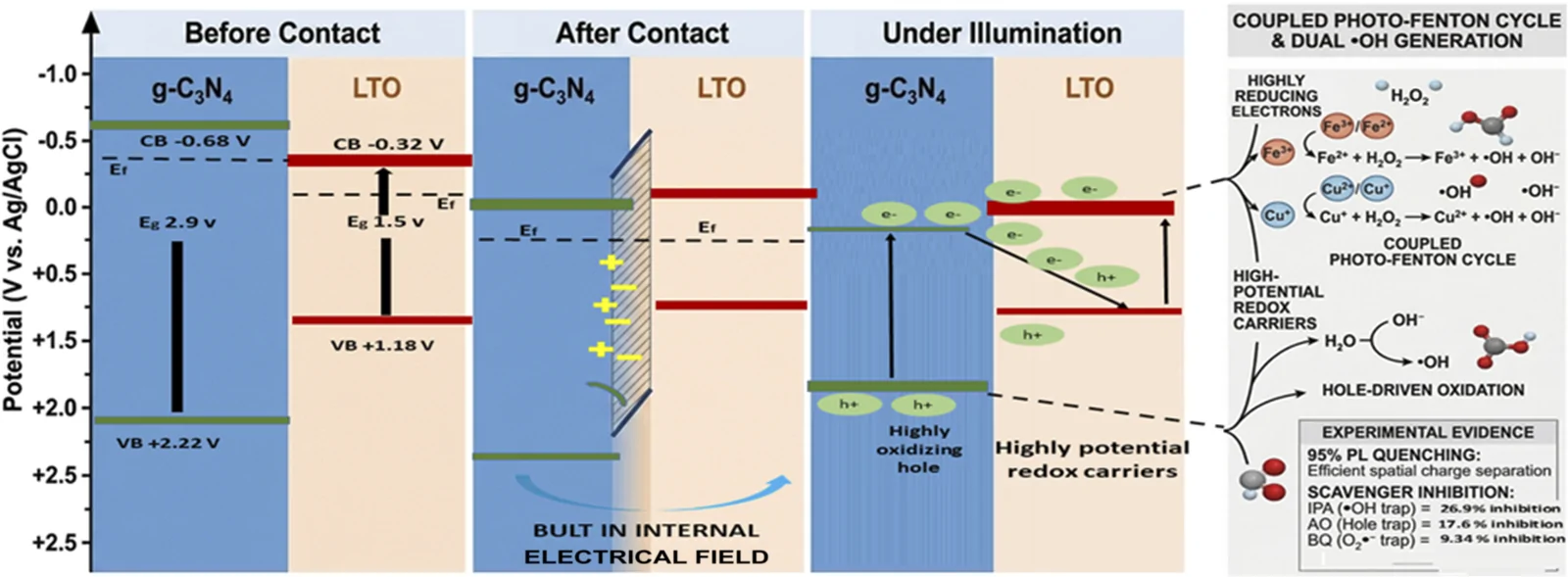
Proposed solid S-scheme mechanism of GLTO.

### 3.4 Degradation pathway of rhodamine B based on LC-MS-MS analysis

The degradation pathway of RhB was investigated by LC-MS analysis of the reaction mixture after 28 min of visible-light photo-Fenton treatment with GLTO, as shown in Fig. 11 and 12. The total-ion chromatograms (TICs) (Fig. 11a and b) and the corresponding mass spectra (Fig. 11c–f) reveal a series of transformation products. The characteristic molecular ion of intact RhB (*m*/*z* 479.2) is absent (*t*_R_ = 1.85 min, area 0.72%), indicating substantial removal of the parent dye from the analyzed reaction mixture. The detected intermediates are consistent with *N*-deethylation and/or decarboxylation of the RhB structure. A prominent signal at *m*/*z* 330.95 (*t*_R_ = 5.15 min, area 16.65%) is tentatively assigned to a deethylated product, while a minor ion at *m*/*z* 393.9 (*t*_R_ = 1.85 min) is tentatively assigned to a decarboxylated species. A signal at *m*/*z* 242.91 (*t*_R_ = 5.87 min, area 12.2%, peak 6) is tentatively assigned to a benzophenone-type structure that may result from oxidative ring opening of the xanthene system. Further transformation is indicated by the lower-molecular-weight ions at *m*/*z* 180.91 (*t*_R_ = 9.81 min, area 10.0%), *m*/*z* 172.1 (*t*_R_ = 2.96 min), and *m*/*z* 112.87 (*t*_R_ = 11.42 min). The progressive decrease in molecular mass, together with the 65% TOC reduction, indicates substantial removal of organic carbon but does not represent complete mineralization. Overall, the proposed pathway involves *N*-deethylation, chromophore cleavage, ring opening, and progressive oxidation toward lower-molecular-weight products. This proposed pathway is consistent with the ˙OH-dominated degradation process indicated by the scavenger experiments and supported by the ESR results

**Fig. 11:**
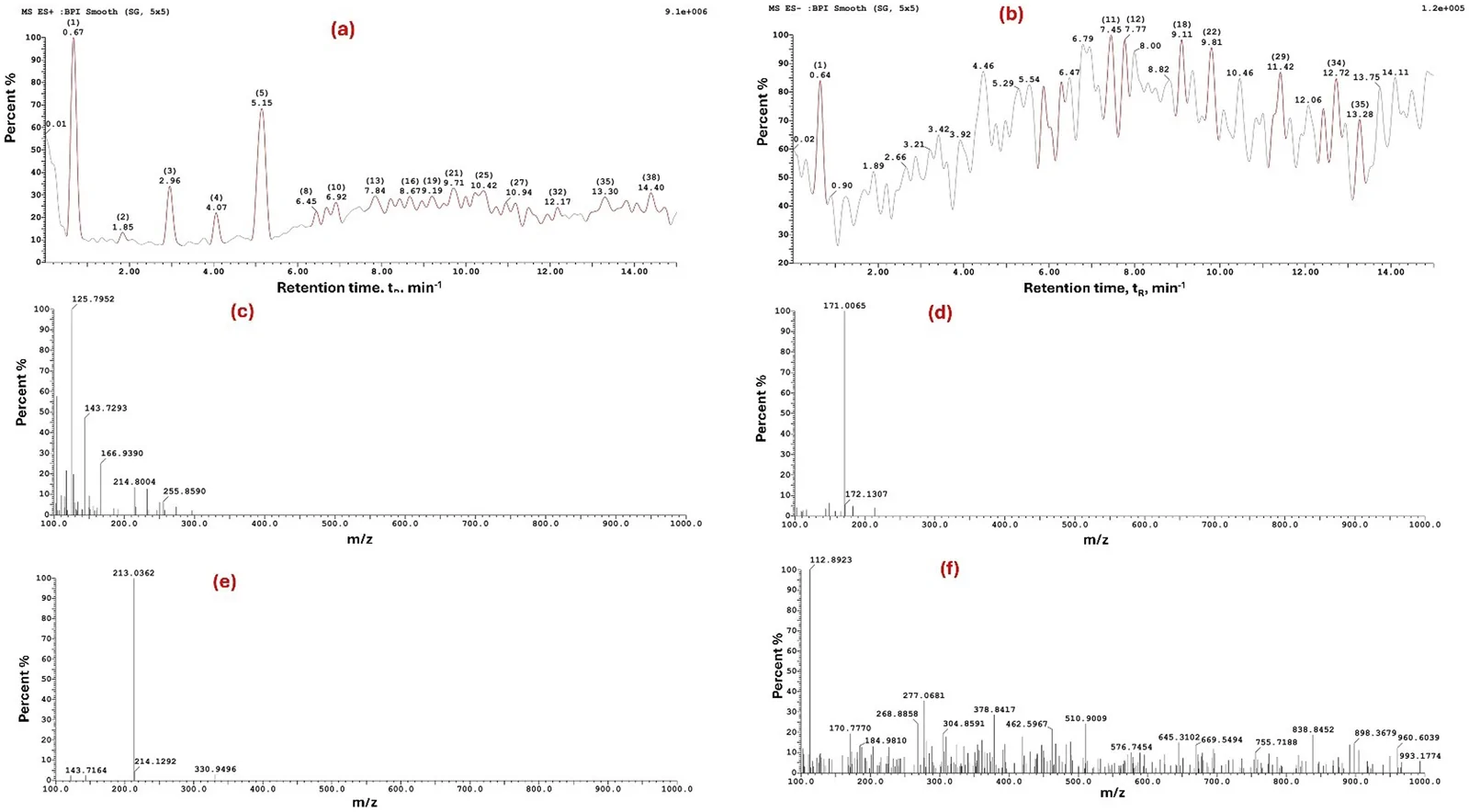
(a and b) Total ion chromatograms (TIC) of the reaction mixture after 28 min of visible-light photo-Fenton degradation of Rhodamine B with GLTO, recorded in positive (ESI^+^) and negative (ESI^−^) modes; (c–f) representative mass spectra of the major degradation intermediates identified at different retention times.

**Fig. 12:**
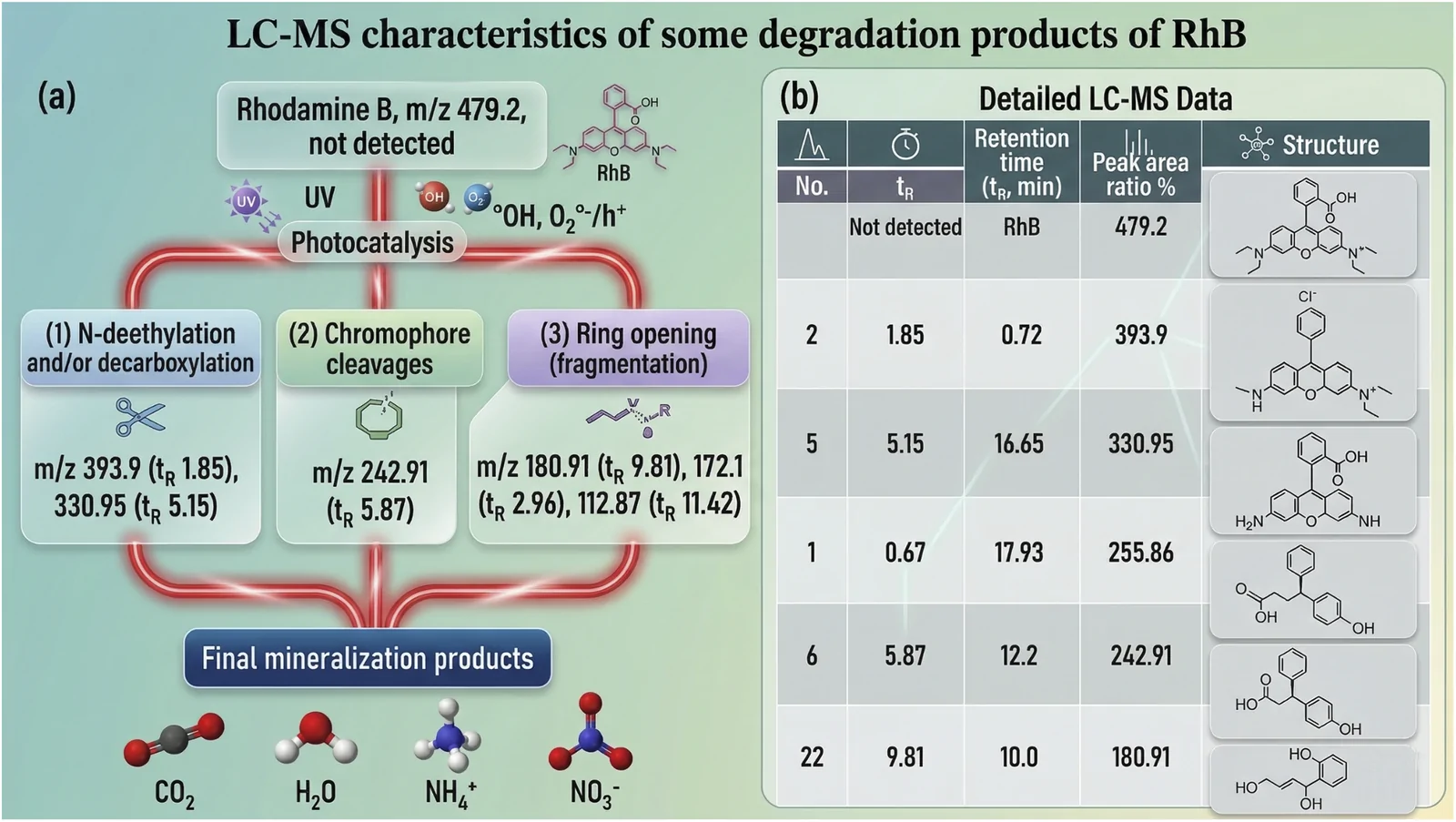
(a) Proposed degradation pathway of RhB; (b) summary table of the main LC-MS peaks.

### 3.5 Toxicity assessment of degradation intermediates

The ECOSAR toxicity assessment provides insight into the predicted environmental hazards associated with the RhB degradation intermediates generated during the GLTO/Vis/H_2_O_2_ process (Table 2). As degradation proceeds from early *N*-deethylated intermediates through chromophore cleavage products to ring-opened species, the predicted toxicity generally decreases. The detected intermediates were tentatively assigned based on the LC-MS data, and their predicted toxicity was evaluated using ECOSAR. Intermediates retaining aromatic structures exhibit acute LC_50_ values for fish, daphnia, and green algae that are predominantly classified as “not harmful” under the criteria applied in this study, although one transient intermediate shows borderline “harmful” effects toward daphnia and algae. The lower-molecular-weight end products are predicted to exhibit lower toxicity. The chronic toxicity values (ChV) reported for the modeled intermediates also remain within the “not harmful” category under the applied ECOSAR assessment. These results indicate a predicted progressive reduction in ecotoxicity as degradation proceeds; however, they should not be interpreted as experimental confirmation of environmental safety or complete detoxification. Experimental ecotoxicity assessment of the degradation products was not performed because the structures of the degradation intermediates were tentatively assigned from the LC-MS data and continuously change as the reaction proceeds.

**Table 2:** ECOSAR-computed toxicity of RhB degradation products (as neutral organics)^a^

SMILES KEY	*m*/*z*	Fish LC50 (96 h)	Daphnia LC50 (48 h)	Green-algae EC50 (96 h)	Fish (ChV)	Daphnia (ChV)	Green algae (ChV)	Earthworm
O3c1cc(ccc1C(c2ccccc2)=C4C=CC(C=C34)=[N+](CC)CC)NC	393.9	7590	3730	1520	625	243	289	905
O=C(O)c1ccccc1C3=C4C=CC(=N)C=C4(Oc2cc(N)ccc23)	330.95	477	292	300	51.1	35.4	93.5	4700
O=C(O)CCC(c1ccccc1)c2ccc(O)cc2	255.86	113	73	93.4	12.9	10.2	32.7	3180
O=C(O)CC(c1ccccc1)c2ccc(O)cc2	242.91	294	182	193	31.8	22.5	61.1	3380
Oc1ccccc1C(O)C=CCO	180.91	2080	1050	480	177	73.8	96.7	425
O=C(O)C(CC)C(C(=O)C)CC	172.1	5680	3030	1750	516	249	398	3520
O=C(C=CC(=O)C)C	112.87	1760	875	378	147	59.1	73.8	274

a Based on the directions of the European Union (Directive 67/548/EEC, Annex VI), a substance is (a) not harmful when LC_50_ > 100 mg L^−1^, (b) harmful when 10 ≤ LC_50_ ≤ 100 mg L^−1^, (c) toxic when 1 ≤ LC_50_ < 10 mg L^−1^, and (d) very toxic when LC_50_ < 1.0 mg L^−1^. Also, based on the Chinese hazard evaluation guidelines for new chemical substances (HJ/T 154–2004) a substance is (a) not harmful when ChV > 10, (b) harmful when 1 ≤ ChV ≤ 10, (c) toxic when 0.1 ≤ ChV < 1, and (d) very toxic when ChV < 0.1.

### 3.6 Comparative performance analysis

The photo-Fenton performance of GLTO was compared with selected recently reported catalysts, as summarized in Table 3. The apparent rate constant obtained for GLTO was 0.0876 min^−1^, compared with 0.0195 min^−1^ for α-Fe_0_._9_/C_3_N_4_/SiO_2_,^35^ 0.0170 min^−1^ for Fe-doped TiO_2_/SiO_2_-OVs,^36^ 0.0225 min^−1^ for NH_2_-MIL-53(Fe),^37^ and 0.0414 min^−1^ for Co_3_O_4_–Bi_2_O_3_–Na_2_S.^39^ A higher value of 0.506 min^−1^ was reported for α-Fe_3_O_4_.^38^ However, the reported values were obtained under different experimental conditions, including variations in catalyst dose, pollutant concentration, H_2_O_2_ concentration, light source, irradiation time, irradiance, and reactor configuration. Therefore, the rate constants listed in Table 3 are provided for comparison with the reported literature data, but direct quantitative comparison should be treated with caution.

**Table 3:** Performance comparison of GLTO with other materials for RhB mineralization under varying conditions

Catalyst	Preparation methods	Wight	Irradiation time	Light source	Mechanism/oxidant/activator	Mechanism	Rate constant (min^−1^)	Band gab	Degradation efficiency	Key findings	Ref.
GLTO	Ultrasonic-assisted assembly with exfoliated g-C_3_N_4_ nanosheets	0.02	28	Sun simulater	Photo-Fenton process with H_2_O_2_ (3 mM)	S-scheme	0.0877	2.4	94%	• S-scheme heterojunction (internal electric field at g-C_3_N_4_/LTO interface drives selective recombination of weaker carriers, preserving strong reducing electrons in g-C_3_N_4_ and oxidizing holes in LTO)	Our work
										• Complete mineralization *via N*-deethylation → ring opening → aliphatic acids	
										• ECOSAR: all intermediates non-toxic or transiently harmful	
ZnFO1/B1 (ZnFe_2_O_4_ : Biochar = 1 : 1)	Sol–gel method	0.1	60	Visible light lamp & natural solar light	H_2_O_2_ (50 µL)	Heterogeneous photo-Fenton	N.D	1.2	100&	• Supporting ZnFe_2_O_4_ on biochar reduced the band gap energy (from 1.67 to 1.12 eV for ZnFO1/B1), which enhances visible light absorption and reduces electron–hole recombination	34
										• Negligible Zn leaching; Fe leaching (1.01 mg L^−1^) below regulatory limits	
										• Higher ferrite ratio may cause particle aggregation or poor distribution on biochar, reducing activity	
α-Fe_2_O_3_/g-C_3_N_4_/SiO_2_	Thermal polycondensation (for g-C_3_N_4_) + in-situ simple precursor drying-calcination process	0.06	120	Simulated visible light (LED lamp, 100 W, *λ* = 420 nm)	H_2_O_2_ (7 × 10^−4^ M)	S-scheme	0.0195	1.5	97%	• The S-scheme heterojunction between α-Fe_2_O_3_ and g-C_3_N_4_ (with SiO_2_ as support) effectively separates charge carriers and maintains high redox potential	35
										• The catalyst showed ferromagnetic properties (Ms = 22 emu g^−1^), allowing easy magnetic separation	
										• DFT calculations and GC-MS analysis proposed a detailed degradation pathway of RhB, including *N*-deethylation and ring-opening steps	
										• SiO_2_ acts as a mesoporous support, increasing surface area and providing a platform for heterojunction formation	
Fe-doped TiO_2_/SnO_2_-OVs (TFS-7)	Hydrothermal method (Fe-doped TiO_2_) followed by hydrothermal heterojunction formation with SnO_2_ (Sn/Ti mol ratio 1/10)	0.025	180	Visible light (Osram lamp 250 W, *λ* ≥ 420 nm, 90 mW cm^−2^)	H_2_O_2_ (12.5 mM optimal)	S-scheme	0.01703	2.22	95%	• Oxygen vacancies (OVs) in SnO_2_ were confirmed by EPR (*g* = 2.002 signal) and play a key role in narrowing bandgap and enhancing charge separation	36
										• DFT calculations showed work functions of 5.549 eV (Fe–TiO_2_) and 7.736 eV (SnO_2_-OVs), driving interfacial charge transfer	
NH_2_-MIL-53(Fe) (amino-functionalized iron-based MOF)	Hydrothermal method (FeCl_3_·6H_2_O + 2-aminoterephthalic acid in DMF at 150 °C for 24 h)	0.008	120	Visible light	H_2_O_2_ (14 mM optimal)	Heterogeneous photo-Fenton	0.0225	N.D	95%	• Amino functionalization improved activity compared to non-amino MIL-53(Fe) (84%), due to better visible light response and reduced electron–hole recombination	37
										• Low iron leaching (0.57 mg L^−1^), below EU environmental standard (2.0 mg L^−1^)	
										• MS analysis identified 10 intermediates; degradation pathways include *N*-demethylation, deamination, decarboxylation, and ring-opening	
α-Fe_2_O_3_ nanoparticles	Electrochemical method	0.1	12	High-pressure mercury lamp (250 W)	H_2_O_2_ (0.36 mmol in 100 mL = 7.2 mM)	Heterogeneous photo-Fenton-like	0.506	1.87	99%	• Oxygen vacancies on the surface create Fe^2+^/Fe^3+^ redox pairs (Fe^2+^ : Fe^3+^ ratio = 1.27), enhancing catalytic activity	38
										• XPS confirmed oxygen vacancies (O 1s peak at 531.7 eV) and Fe^2+^ presence (710.1 and 724.5 eV)	
CoNiO_2_–BiFeO_3_–NiS	Co-precipitation + calcination	0.004	100	Visible light	H_2_O_2_	Z-scheme	0.0414	2.35	97.8	• GC-MS identified degradation intermediates; TOC confirmed high mineralization (97% for MO, 96% for RhB)	39
										• ECOSAR toxicity assessment predicted end products as non-toxic – unique feature	
										• Multiferroic properties (BiFeO_3_) enable magnetic separation	

The GLTO composite achieved 93.5% removal of RhB within 28 min under the experimental conditions used in this study. Reported removal efficiencies for α-Fe_3_O_4_ (ref. 38) and ZnFO1/B1 (ref. 34) were 99% and 100%, respectively, although different irradiation times and reaction conditions were used. The GLTO experiments were conducted using 0.02 g of catalyst and 3 mM H_2_O_2_. These values represent the catalyst and oxidant concentrations used in the present experiments and provide additional parameters for comparison with the reported systems.

The proposed S-scheme charge-transfer configuration provides a possible explanation for the charge-transfer behavior of the GLTO composite. In this model, electrons are retained on the g-C_3_N_4_ side, while holes are retained on the LTO side. The PL, EIS, ESR, band-position analysis, and scavenger experiments provide complementary evidence that is consistent with this proposed charge-transfer pathway; however, these results do not independently establish the S-scheme mechanism. The LC-MS results tentatively indicate progressive transformation of RhB through *N*-deethylation, chromophore cleavage, ring opening, and formation of lower-molecular-weight products. The 65% TOC reduction indicates a substantial decrease in the organic carbon content of the solution but does not indicate complete mineralization. ECOSAR analysis provides a predictive assessment of the potential toxicity of the tentatively assigned intermediates rather than experimental confirmation of their environmental effects. Under the tested model water conditions, RhB removal reached approximately 90% at 10 mM NaCl and 72% at 10 mg L^−1^ humic acid. After five consecutive cycles, approximately 95% of the initial degradation efficiency was retained. These results describe the performance of GLTO under the specific experimental conditions investigated in this study.

## 4. Conclusion

This study demonstrates that coupling LTH-derived multimetal oxides (LTO) with exfoliated g-C_3_N_4_ produces a visible-light-active heterojunction (GLTO) with enhanced charge separation and photo-Fenton performance. The proposed S-scheme model is consistent with preservation of strongly reducing electrons in the g-C_3_N_4_ conduction band and oxidizing holes on the LTO side, while interfacial charge transfer may help suppress recombination. The retained electrons can promote O_2_ activation and Fe^3+^/Cu^2+^ reduction, thereby sustaining Fe/Cu-mediated H_2_O_2_ activation and ˙OH generation. Oxidizing holes on the oxide side can contribute to direct oxidation of adsorbed organic species. These coupled pathways provide a mechanistic basis for the higher activity of GLTO than that of the individual components.

In addition to its contribution to charge separation, the mutual dispersion of LTO nanoparticles and g-C_3_N_4_ nanosheets produces a hierarchical mesoporous architecture that improves accessible surface area and reactant transport. GLTO achieved 93.5% RhB removal and 65% TOC reduction, retained approximately 90% degradation efficiency at 10 mM NaCl and 72% at 10 mg L^−1^ humic acid under the tested model water conditions, and maintained approximately 95% of its initial activity after five reuse cycles. LC-MS analysis tentatively indicates progressive degradation and the formation of degradation intermediates, while ECOSAR modeling predicts a reduction in the potential toxicity of the detected intermediates. These results demonstrate the applicability of LTH-derived multimetal oxides as components for constructing g-C_3_N_4_-based photo-Fenton heterojunctions. Future work should evaluate alternative LTH compositions, experimentally verify the toxicity of treated effluents, quantify metal leaching and energy consumption, and assess performance in continuous-flow systems and real wastewater containing broader classes of recalcitrant contaminants.

## Conflicts of interest

There are no conflicts to declare.

## Supplementary Material

RA-OLF-D6RA06898G-s001

## Data Availability

All data supporting the findings of this study are included in the main manuscript and the supplementary information (SI). Supplementary information is available. See DOI: https://doi.org/10.1039/d6ra06898g.
