# Supplementary material for: S-Scheme charge transfer in topotactically transformed LTO/g-C3N4 for photo-Fenton mineralization in complex water matrices
Source: RSC Adv. 2026 Oct 14;16(47):62814–36. doi: 10.1039/d6ra06898g (PMC13662861; doi:10.1039/d6ra06898g)
Supplement: Supplementary file 1 [file RA-016-D6RA06898G-s001.pdf]

Supplementary Information (SI) for RSC Advances.
This journal is © The Royal Society of Chemistry

S-Scheme Charge Transfer in Topotactically Transformed LTO/g-C_3N_4 for Photo-Fenton Degradation in Complex Water Matrices

Fowzia S. Alamro[1], Wafaa B. Elsharkawy [2], Arafat Toghan[3], Ashraf A. Mohamed[4], Ayman M. Mostaf[5], Mohamed Farag[4], Hoda A. Ahmed[5] , Mohamed A. Ahmed[4], Mahmoud A. Elsayed*

[1] Department of Chemistry, College of Science, Princess Nourah bint Abdulrahman University, P.O. Box 84428, Riyadh 11671, Saudi Arabia
[2] Physics Department, College of Science and Humanities, Prince Sattam Bin Abdulaziz University, Al-Kharj 11942, Saudi Arabia
[3] Chemistry Department, College of Science, Imam Mohammad Ibn Saud Islamic University (IMSIU), 11623, Riyadh, Saudi Arabia
[4]Chemistry department, Faculty of Science, Ain Shams University, Cairo-11566, Egypt
[5]Chemistry Department, Faculty of Science at Yanbu, Taibah University, Yanbu 46423, Saudi Arabia

*Corresponding author:

Mahmoud. A. Elsayed (Mahmoud.a.elsayed1552@gmail.com)

Materials and Reagents

Analytical-grade chemicals were used throughout all experiments without any subsequent purification. High-purity urea served as the sole nitrogen precursor for synthesizing the graphitic carbon nitride (g-C₃N₄) matrix. To fabricate the layered triple hydroxide (LTH) precursor—which was subsequently transformed into the ternary oxide heterojunction—zinc acetate dihydrate, copper(II) sulfate pentahydrate, and iron(III) chloride were employed as the Zn, Cu, and Fe sources, respectively. Coprecipitation and alkaline pH conditions were strictly maintained using sodium hydroxide (NaOH, ≥98%) and sodium carbonate (Na₂CO₃, 99.5%). Hydrogen peroxide (H₂O₂, 30% w/w) was utilized as the primary oxidant in the visible-light-driven photo-Fenton experiments targeting the degradation of the model pollutants: Rhodamine B (RhB) and Indigo Carmine (IC, C.I. 73015). To elucidate the degradation mechanism and identify the dominant reactive species, isopropanol (IPA, 99.5%), p-benzoquinone (p-BQ, ≥98%), and ammonium oxalate (AO) were introduced as selective trapping agents for hydroxyl radicals (•OH), superoxide radicals (•O₂⁻), and photogenerated holes (h⁺), respectively. Absolute ethanol and ultrapure deionized water were applied as

solvents during both the synthetic procedures and the ultrasonic exfoliation steps. Finally, technical-grade humic acid (HA) and sodium chloride (NaCl, 99.5%) were selected to simulate natural organic matter and salinity, respectively, to evaluate the catalyst performance under model water conditions.

Materials characterization

Fourier-transform infrared (FTIR) spectra were acquired using a KBr disk technique on an ATI Mattson FTIR spectrometer. High-resolution transmission electron microscopy (HRTEM) was employed to capture detailed images, utilizing a JEOL-2100-HRTEM instrument. X-ray photoelectron spectra were recorded using a K-ALPHA-XPS instrument (Thermo Fisher Scientific, USA) with monochromatic X-ray Al Kα radiation ranging from 10 to 1350 eV. Diffuse reflectance spectroscopy measurements were conducted using a V-570-JASCO spectrometer. The FLS1000 Photoluminescence Spectrometer (Edinburgh Instruments, UK) was used for PL measurements with a 380 nm excitation source. Absorption measurements were performed in 10 mm matched cuvettes using a Shimadzu-1650 UV/VIS absorption spectrophotometer (Kyoto, Japan) controlled by UV-probe 2.5 software.

Total organic carbon (TOC) measurements were performed to evaluate the mineralization degree of Rhodamine B following the photo-Fenton reaction. Water samples collected immediately after the completion of the reaction (at 28 min) were filtered through 0.22 μm syringe membrane filters to completely remove any suspended catalyst particles. The filtrate

was then analyzed using a TOC analyzer to determine the residual organic carbon content, and the mineralization efficiency was calculated by comparing the final TOC value with the initial TOC of the dye solution.

The surface charge characteristics and the point of zero charge (pHpzc) of the GLTO composite were determined using a zeta potential analyzer. In a typical measurement, 10 mg of the catalyst powder was dispersed in 50 mL of an aqueous solution containing 0.1 mM NaCl as a background electrolyte. The pH of the suspension was adjusted across a range from 2 to 10 using dilute HCl and NaOH solutions. The suspensions were magnetically stirred to ensure thorough dispersion and equilibrium before measurement. The zeta potential values were recorded at room temperature as a function of pH.

Electrochemical measurements, including galvanostatic electrochemical impedance spectroscopy (EIS) and Mott-Schottky (M-S) analysis, were performed using a CorrTest electrochemical workstation (CS310X) equipped with a standard three-electrode cell configuration. The prepared samples coated on conductive substrates served as the working electrode with an exposed active area of 1 cm^2. An Ag/AgCl electrode was used as the reference electrode, and a graphite rod was utilized as the counter electrode. All electrochemical tests were conducted in a 0.5 M Na_2SO_4 aqueous electrolyte. The galvanostatic EIS measurements were carried out at an applied direct current (DC) of 0 mA with an alternating current (AC) amplitude of 50 mA, scanning over a frequency range from 50 kHz down to 0.01 Hz (collecting 67 data points). The M-S measurements were conducted

using a sinusoidal AC perturbation with a voltage amplitude of 10 mV at a constant frequency of 1000 Hz. The applied potential was swept from −0.6 V to +1.5 V vs. Ag/AgCl.

Electron paramagnetic resonance (EPR) measurements were conducted to characterize paramagnetic species associated with the catalyst samples. The EPR spectra were recorded at room temperature using an X-band EMX spectrometer (Bruker, Germany) equipped with a standard rectangular cavity (ER 4102) and a Klystron microwave generator operating at a frequency of approximately 9.5 GHz. In a typical procedure, the samples were placed in quartz capillary tubes, and the measurements were carried out using a modulation frequency of 100 kHz. The magnetic field at the resonance peak (H₀), peak linewidth (ΔH), and signal intensity (A) were recorded for comparison of the EPR characteristics of the catalyst samples.

Liquid chromatography-mass spectrometry (LC-MS) analysis was performed to identify the degradation intermediates of RhB. The analysis was conducted using a Waters ACQUITY UPLC system coupled with a XEVO-TQD triple quadrupole mass spectrometer (Waters, USA). Chromatographic separation was carried out at a flow rate of 0.200 mL/min with an injection volume of 7 μL. A gradient elution program was applied over a 15-minute run time, utilizing water containing 0.1% formic acid as the primary aqueous phase. The mass spectrometer was equipped with an electrospray ionization (ESI) source, operated in both positive (ES+) and negative (ES−) ionization modes. The full-scan mass spectra were acquired over an m/z range of 100 to 1000. The mass spectrometry parameters were set as follows: capillary voltage of 3.05 kV for ES+ and 3.00 kV for ES−, cone voltage of 35.41 V, source

temperature of 150 °C, desolvation temperature of 400 °C, desolvation gas flow of 600 L/h, and cone gas flow of 50 L/h.

The predictive environmental toxicity of RhB and its tentatively identified degradation intermediates was evaluated using the Ecological Structure Activity Relationships (ECOSAR) predictive model software (v2.2, US EPA). The Simplified Molecular Input Line Entry System (SMILES) notations of the proposed intermediate structures were used as input data for the software. The model estimated the acute toxicity (LC₅₀ for fish and daphnia, EC₅₀ for green algae) and chronic toxicity values (ChV) for the tentatively assigned intermediates.

Experimental Section: Adsorption and Photocatalytic Degradation

The adsorption kinetics and visible-light-driven photo-Fenton activity of the GLTO heterojunction were evaluated using Rhodamine B (RhB) as the primary model pollutant. In a typical batch experiment, 0.02 g of the catalyst was dispersed in 100 mL of an aqueous RhB solution with an initial concentration of 10 mg L⁻^{1} at an initial pH of 7. The suspension was magnetically stirred in the dark for 30 minutes to achieve adsorption-desorption equilibrium. The equilibrium adsorption capacity (qₑ, mg g⁻^{1}) was calculated using Eq. (1): qₑ = (C₀ − Cₑ)V/m, where C₀ and Cₑ represent the initial and equilibrium concentrations (mg L⁻^{1}), V is the solution volume (L), and m is the catalyst mass (g). To evaluate the adsorption kinetics, the experimental dark adsorption data were fitted to the pseudo-first-order (PFO), pseudo-second-order (PSO), and intraparticle diffusion (Weber–Morris) kinetic models. The PFO and PSO

models were fitted using nonlinear regression in Origin software, whereas the Weber–Morris model was evaluated by linear fitting of q_t versus $t^{0.5}$.

Subsequent photo-Fenton degradation experiments were conducted under visible-light irradiation in a thermostated reactor maintained at a constant temperature of 30 °C. The optimal concentration of H_2O_2 (3 mM) was introduced into the reaction mixture. Visible-light illumination was provided by a 300 W Xenon solar simulator equipped with a UV cut-off filter ($\lambda \geq 420$ nm), with a fixed lamp-to-reactor distance of 30 cm. Aliquots were periodically withdrawn, filtered, and analyzed spectrophotometrically over a 28-minute reaction period to monitor the degradation process.

To identify the dominant reactive oxygen species driving the proposed S-scheme mechanism, specific radical scavengers were introduced prior to irradiation: isopropanol (IPA) as a hydroxyl radical (•OH) trap, p-benzoquinone (BQ) as a superoxide radical ($\bullet O_2^-$) trap, and ammonium oxalate (AO) as a photogenerated hole (h^+) trap.

Performance in Model Water Matrices and Reusability

To evaluate the performance of the catalyst under model water conditions, the photo-Fenton degradation of RhB was examined in model water matrices. This included the addition of sodium chloride (NaCl, at concentrations up to 10 mM) to simulate salinity effects, and humic acid (HA, at concentrations up to 10 mg L^{-1}) to mimic natural organic matter. Additionally, the influence of the initial solution pH was examined at pH 4, 6, and 8 to evaluate the effect of

pH on the catalyst performance. Finally, the recyclability of the GLTO composite was assessed through five consecutive photo-Fenton degradation cycles under identical experimental conditions, with the catalyst being recovered, washed, and dried between cycles.
